# Occupational mobility and automation: a data-driven network model

**DOI:** 10.1098/rsif.2020.0898

**Published:** 2021-01-20

**Authors:** R. Maria del Rio-Chanona, Penny Mealy, Mariano Beguerisse-Díaz, François Lafond, J. Doyne Farmer

**Affiliations:** 1 Institute for New Economic Thinking at the Oxford Martin School, University of Oxford, Oxford, UK; 2 Mathematical Institute, University of Oxford, Oxford, UK; 3 School of Geography and Environment, University of Oxford, Oxford, UK; 4 Smith School of Environment and Enterprise, University of Oxford, Oxford, UK; 5 Soda Laboratories, Monash Business School, Monash University, Clayton, Australia; 6 Santa Fe Institute, Santa Fe, New Mexico, USA

**Keywords:** networks, automation, labour market, unemployment, agent-based model

## Abstract

The potential impact of automation on the labour market is a topic that has generated significant interest and concern amongst scholars, policymakers and the broader public. A number of studies have estimated occupation-specific risk profiles by examining how suitable associated skills and tasks are for automation. However, little work has sought to take a more holistic view on the process of labour reallocation and how employment prospects are impacted as displaced workers transition into new jobs. In this article, we develop a data-driven model to analyse how workers move through an empirically derived occupational mobility network in response to automation scenarios. At a macro level, our model reproduces the Beveridge curve, a key stylized fact in the labour market. At a micro level, our model provides occupation-specific estimates of changes in short and long-term unemployment corresponding to specific automation shocks. We find that the network structure plays an important role in determining unemployment levels, with occupations in particular areas of the network having few job transition opportunities. In an automation scenario where low wage occupations are more likely to be automated than high wage occupations, the network effects are also more likely to increase the long-term unemployment of low-wage occupations.

## 1. Introduction

In response to widespread concern about the potential impact of automation on the labour market [1–5], significant effort has been devoted towards analysing how susceptible a given occupation is to computerization [6–8]. However, studies that estimate the likelihood of a robot ‘stealing’ a particular job only provide part of the picture. Consider, for example, the job security faced by a statistical technician vs. a childcare worker. Estimates developed by Frey and Osborne [8] suggest that statistical technicians are more likely than childcare workers, for example, to be replaced by software technology. However, should such forecasts eventuate, and statistical technicians find themselves out of a job, their existing skills could allow them to transition into a range of ‘safer’ occupations with lower automation risk and growing demand. In contrast, while childcare workers may not experience a *direct* threat from computerization, their employment prospects may nonetheless still be impacted. As automation displaces people in other occupations, many of these workers could have the requisite skills to become childcare workers and may consequently provide an *indirect* effect on the job security of existing childcare workers. Thus, even though the immediate risk of automation is predicted to be larger for statistical technicians, accounting for possible occupational transitions and labour demand reallocation and could see childcare workers facing a greater risk of unemployment. To study these important, but overlooked indirect labour displacement effects, this article develops a new data-driven model of the labour market.

There is a rich body of literature that has demonstrated the importance of modelling labour flows using agent-based models and networks. Reference [9] surveyed several agent-based models; some of these models can be used to test different labour market policies. Reference [10] proposed an agent-based model for the French labour market, and references [11–16] used networks to model how workers move between industries and firms. These models can help understand the first-order displacement effects of labour market shocks better. Networks have also been used to better understand how skills, knowledge, and work activities are distributed across occupations [17–19]. We build on this body of work and go further by modelling the labour market out of equilibrium and considering indirect displacement effects of automation. Central to our model is an empirically derived *occupational mobility network*, in which nodes are different occupations and edges correspond to the probability that workers transition between them. The overall structure of this network influences the efficiency with which workers are reallocated across occupations following a shift in relative labour demand.

To explore the potential impacts of automation on the labour market, we impose an automation ‘shock’ that, over the years, decreases the demand for labour in some occupations and increases demand in others. By using an agent-based model, we study the associated aggregate and occupation-specific unemployment dynamics as a function of time. While we analyse the results for only two automation shock scenarios based on estimates developed by Frey and Osborne [8] and Brynjolfsson *et al.* [7], our model is general and can be used to study a range of different labour market shocks.

We model the resulting process of labour reallocation as a stochastic process with discrete time steps. During each time step, job vacancies of different occupations open, and some workers are separated (fired), unemployed workers apply for a new job, and vacancies are matched with job applicants. We model an out-of-equilibrium economy and focus on the transient dynamics during which the labour market re-adjusts to a new steady state. In addition to simulations, we also derive a representation of the model as a deterministic dynamical system that, in the limit of a large number of workers, predicts the expected behaviour of the model simulations. This provides deeper insights into the mechanics of the model and dramatically speeds up computations with little accuracy loss.

We assume that the relative labour demand between occupations changes due to automation, but the total demand for jobs across occupations is constant. We focus on a transition period of around a decade right after the automation shock hits, and the bulk of labour reallocation occurs. We analyse the impacts on both short-term and long-term unemployment (> 27 weeks). Unsurprisingly, we find that occupations at higher risk of automation tend to be affected most, but we also show that restrictions on worker movements imposed by the occupational mobility network generate substantial labour market mismatch. In some areas of the network, many workers can be competing for a small number of vacancies. Simultaneously, occupations in other areas can have vacancies that are left unfilled for a long time. Compared to a labour market with no mobility restrictions, the occupational mobility network structure increases unemployment by roughly 29%. We also show that occupations with the same level of *ex ante* automation risk can end up with markedly different unemployment levels.

Our model also provides insights into the Beveridge curve, a well-known negative empirical relationship between the unemployment rate and the vacancy rate [20]. Typically, when vacancies increase, unemployment goes down. We show that after parameter calibration, our model reproduces the empirical Beveridge curve during the most recent US business cycle and supports the hypothesis that business cycles alone can cause the anticlockwise cycling behaviour of the curve [21–23].

Our results have important implications for designing policies aimed at helping workers best prepare and adapt to the changing nature of the labour market. More nuanced insights into employment impacts associated with automation could help improve the effectiveness of worker retraining schemes. For example, rather than only considering workers’ current occupation’s susceptibility to automation, skill development programmes could be more efficiently targeted towards workers in occupations that are likely to face longer spells of unemployment [24]. Furthermore, a better understanding of the mechanisms underpinning the Beveridge curve could help policymakers mitigate adverse employment impacts of business cycles and accelerate the recovery process.

## 2. Model design

### 2.1. The occupational mobility network

**Figure 1.**
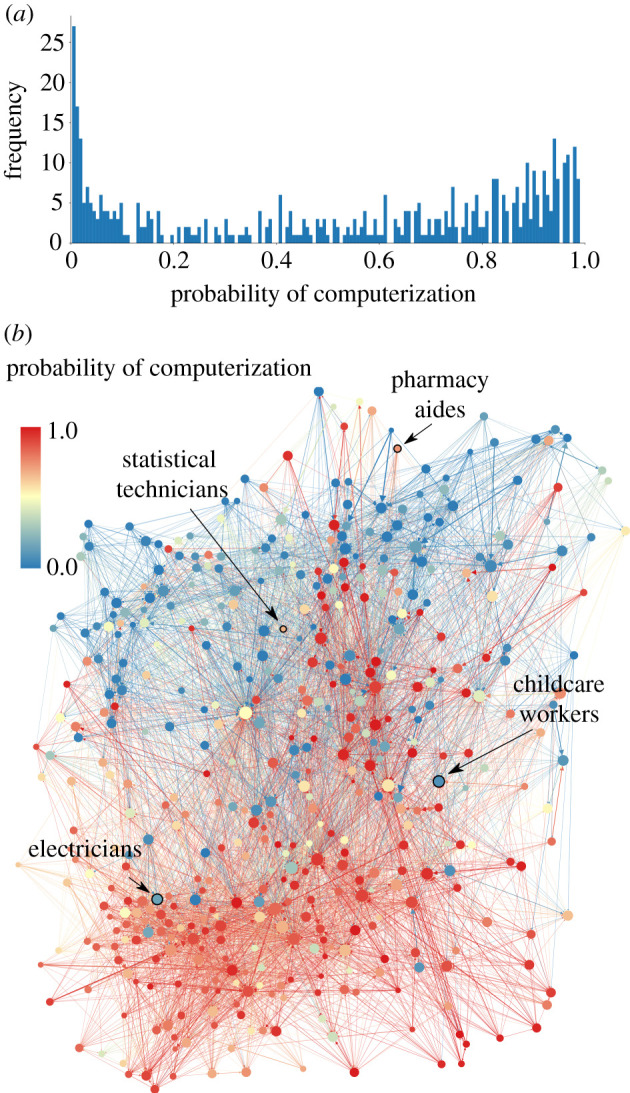
Estimates of automatability in the occupational mobility network. (*a*) A histogram of the probability of computerization for different occupations as estimated by Frey and Osborne [8], suggesting a bimodal distribution. (*b*) The occupational mobility network, where nodes represent occupations and links represent possible worker transitions between occupations. Red nodes have high automatability, and blue nodes have low automatability. The size of the nodes indicates the logarithm of the number of employees in each occupation.

We first construct an occupational mobility network to capture the ease with which a worker can transition between occupations. We follow the work of Mealy *et al.* [19] and construct the network based on the data on occupational transitions in the United States between 2010 and 2017 [25]. In this network, nodes are occupations, and the weights of the edges are proportional to the probability that a worker transitions between occupations. The resulting network is weighted and directed with *n* = 464 nodes (see figure 1*b*). The network also has self-loops since workers often remain in the same occupation when they change jobs. We represent the network by its adjacency matrix *A*, with elements

2.1 $$A_{ij}=\left\{ \begin{array}{cc} r\; & \text{if }\;i=j\;\\ (1-r)P_{ij}\; & \text{if }\;i\neq j,\; \end{array}\right.$$

where the indices *i* and *j* label the *n* possible occupations. *r* is the weight of the self-loops and is the probability that a worker from occupation *i* who changed jobs remains in her same occupation. *P*_*ij*_ is the empirical probability that a worker transitioning out of occupation *i* moves to occupation *j*. In the electronic supplementary material, we provide details on how we compute *P*_*ij*_ section S1.1 and a robustness check in section S4 where we use heterogeneous values of the self-loops. We assume that *A*_*ij*_ is fixed in time—edges do not change, and no nodes are removed or added.

Figure 1*b* shows the occupational mobility network, making it clear that there is a rich structure underlying occupational transitions [19]. As a result, occupational mobility is significantly more restricted than is commonly assumed in the most basic labour market models, as noted in references [26,27]. Figure 1*b* also shows estimates of the automatability of occupations, revealing that while there are clear clusters of high or low automatability, specific occupations have a very different degree of automatability than their neighbouring occupations and will therefore be strongly affected by indirect (reallocation) effects.

### 2.2. A network model of the labour market

Our model is designed to understand the dynamics of unemployment at the occupation level. The flow of workers on the network (i.e. workers changing occupations) is described by a set of discrete-time stochastic processes for employment, unemployment and vacancies in each occupation *i*. The agents in our model are workers who can be employed or unemployed. All workers have only one occupation per time step, but they can switch occupations. In this model, we assume that workers are perfectly geographically mobile, and we neglect wage pressure. The set of possible occupations is fixed, and we define a worker’s occupation as the occupation in which she was last employed. At any given time *t*, the number of workers employed in occupation *i* is *e*_*i*,*t*_, the number of unemployed workers is *u*_*i*,*t*_ and the number of job vacancies is *v*_*i*,*t*_. The number of workers that are separated (i.e. fired) is *ω*_*i*,*t*_, and the number of newly opened vacancies is *ν*_*i*,*t*_. The labour flow *f*_*ij*,*t*+1_ is the number of workers hired in occupation *j* who were previously unemployed in occupation *i*. By using these notations, we can write

2.2 $${\;}_{i,t+1}=e_{i,t}-\underset{\mathrm{separated}\;\mathrm{workers}}{\underbrace{\omega_{i,t+1}}}+\underset{\mathrm{hired}\;\mathrm{workers}}{\underbrace{\sum_{j}f_{\;ji,t+1}}}$$

2.3 $$u_{i,t+1}=u_{i,t}+\underset{\mathrm{separated}\;\mathrm{workers}}{\underbrace{\omega_{i,t+1}}}-\underset{\mathrm{transitioning}\;\mathrm{workers}}{\underbrace{\sum_{j}f_{ij,t+1}}}$$

and

2.4 $$\;v_{i,t+1}=v_{i,t}+\underset{\mathrm{opened}\;\mathrm{vacancies}}{\underbrace{\nu_{i,t+1}}}-\underset{\mathrm{hired}\;\mathrm{workers}}{\underbrace{\sum_{j}f_{\;ji,t+1}}}.$$

**Figure 2.**
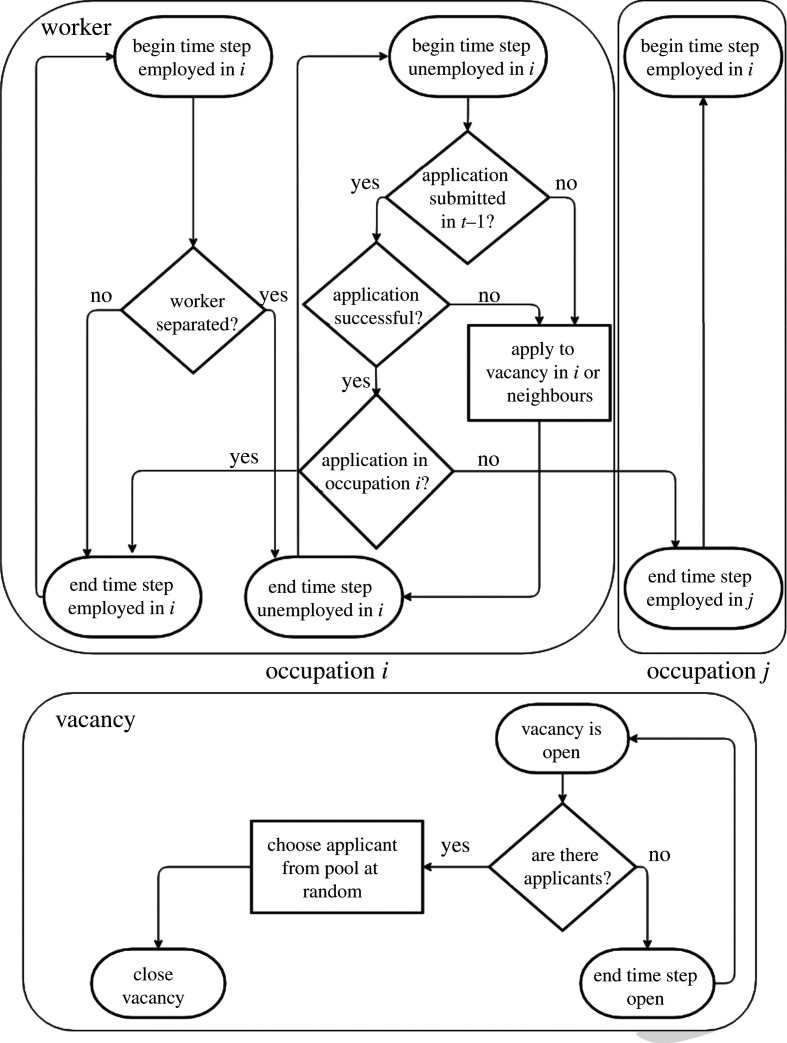
Flow chart illustrating the possible transitions of workers and job vacancies during a given time step. *Top*: Transitions of a worker. *Bottom*: The transitions of a job vacancy. Note that vacancies created in the current time step do not accept job applications until the following time step.

These equations express conservation laws stating that the change in each variable is equal to the difference between inflow and outflow. Equation (2.2) states that the change in employment is equal to the number of workers hired minus the number of workers separated. Similarly, equation (2.3) states that the change in unemployment is equal to the number of separated workers minus the number of hired workers. Finally, equation (2.4) states that the change in the number of vacancies is equal to the number of vacancies created minus the number of workers hired to fill existing vacancies. Figure 2 is a flow chart that makes the transitions explicit from a worker’s perspective and an employer’s perspective.

Our model builds on Mortensen and Pissarides’ studies [28,29], who proposed the use of a matching function between workers and vacancies. In our model, the labour flow *f*_*ij*,*t*+1_ corresponds to a new matching function, which has the advantage of being granular (i.e. it is occupation-specific) and motivated by the stochastic process illustrated in figure 2.

We denote occupation-specific stochastic variables by lowercase letters and aggregate quantities by uppercase letters (e.g. total unemployment is $U_{t}=\sum_{i}u_{i,t}$) and use bold font for vectors (i.e. the *i*-th element of **u**_*t*_ is *u*_*i*,*t*_). We denote realized values of stochastic variables by a hat above e.g. ${\hat{e}}_{i,t}$ is a realization of the stochastic variable *e*_*i*,*t*_. The time steps are chosen, so that their duration is long enough for workers to transition between occupations, but too short for workers to change their employment status more than once. That is, a worker is not allowed to switch her status from employed to unemployed and then back to employed in a single time step. Likewise, a vacancy cannot be opened and filled within the same time step.

We assume that the number of separated workers *ω*_*i*,*t*+1_ and the number of opened vacancies at *t* + 1 *ν*_*i*,*t*+1_ follow binomial processes of the form,

2.5 $$\omega_{i,t+1}∼\text{Bin}({\hat{e}}_{i,t},\pi_{u,i,t})$$

and

2.6 $$\nu_{i,t+1}∼\text{Bin}({\hat{e}}_{i,t},\pi_{v,i,t}),$$

where Bin(*m*, *p*) denotes a binomial distribution with *m* trials and success probability *p*. The success probabilities ${\hat{\pi }}_{u,i,t}$ and ${\hat{\pi }}_{v,i,t}$ depend on ${\hat{d}}_{i,t}-d_{i,t}^{†}$, which is related to the imbalance of supply and demand for labour and play a key role in the dynamics. We will first complete an overview of the search and matching process and return in a moment to specify *π*_*u*,*i*,*t*_ and *π*_*v*,*i*,*t*_.

#### 2.2.1. Search and matching

The labour flow *f*_*ij*,*t*+1_ depends on the occupational mobility network structure, the number of vacancies and unemployed workers and the job search and matching process. We assume that each unemployed worker makes exactly one job application (this facilitates the mathematical derivations). We denote the probability that a worker in occupation *i* applies to a vacancy in occupation *j* by *q*_*ij*,*t*+1_ and assume that *q*_*ij*,*t*+1_ is proportional to the number of vacancies in occupation *j* and the empirical transition probability^1^ between the two occupations *A*_*ij*_. This leads to the following functional form:

2.7 $$q_{ij,t+1}=\frac{{\hat{v}}_{\;j,t}A_{ij}}{\sum_{l}{\hat{v}}_{l,t}A_{il}},$$

where we have the number of vacancies of occupation *j* multiplied by the empirical transition probability *A*_*ij*_ in the nominator and we have a normalizing factor that guarantees that $\sum_{j}q_{ij,t+1}=1$ in the denominator. After all workers have placed their application, one applicant chosen uniformly at random is hired for each vacancy. Some vacancies may not receive applications, in which case no one is hired, and the job vacancy remains open. At the next time step, the model repeats itself as illustrated in figure 2. In the Results section, we present analytical approximations to the dynamics of the model and make more explicit how the flow of workers depends on the occupational mobility network structure.

#### 2.2.2. Supply and demand for labour

Workers move across the occupational mobility network in response to shifts in labour demand. These shifts are determined by the success probability *π*_*u*,*i*,*t*_ of the binomial process for separating workers in equation (2.5) and the success probability *π*_*v*,*i*,*t*_ for the binomial process for creating vacancies in equation (2.6). We break each of these into two separate random processes. The first is a *spontaneous process* (or state independent), and the second is a *state-dependent process*.

In the spontaneous process, workers are separated, and vacancies are opened at random, independent of the state of the system. For simplicity, we assume that the separation and opening rates are the same for all occupations. For any given occupation, the spontaneous probability that a given worker is separated at any given time is *δ*_*u*_, and the spontaneous probability that a vacancy opens is *δ*_*v*_ times the number of workers in that occupation.

The state-dependent process drives the labour demand reallocation by adjusting the *realized* labour demand towards the *target* labour demand. The target labour demand $d_{i,t}^{†}$ is the desired quantity of labour for occupation *i* at time *t*. The target demand is imposed externally and allows us to impose automation shocks (or other reallocation shocks) as a function of time. The realized labour demand, in contrast, is a time-dependent variable corresponding to the sum of the number of employed workers plus the number of job vacancies in a given occupation, i.e.

$$d_{i,t}=e_{i,t}+v_{i,t}.$$

The difference between the realized and the target demand can be attributed to supply factors (e.g. when there are more employed workers in an occupation than the target demand requires) or to demand factors (e.g. when vacancies are scarcer than what the target demand dictates). The separation of workers and opening of job vacancies allow the realized labour demand to adjust: the realized labour demand for an occupation *i* increases when vacancies of occupation *i* open and decreases when workers of occupation *i* are separated. In this state-dependent process, we equilibrate the realized labour demand *d*_*i*,*t*_ to the target demand $d_{i,t}^{†}$ by opening more vacancies if $d_{i,t}<d_{i,t}^{†}$ or separating workers if $d_{i,t}>d_{i,t}^{†}$. To do so, we define an additional occupation-specific probability *α*_*u*,*i*,*t*_ that a worker from occupation *i* is separated at time *t* and an additional occupation-specific probability *α*_*v*,*i*,*t*_ that a vacancy in occupation *i* opens. Both of these probabilities are functions of time and depend on the difference between the realized and target labour demand. We assume the following functional forms

2.8 $$\alpha_{u,i,t}=\gamma_{u}\frac{\max\{0,d_{i,t}-d_{i,t}^{†}\}}{e_{i,t}}$$

and

2.9 $$\alpha_{v,i,t}=\gamma_{v}\frac{\max\{0,d_{i,t}^{†}-d_{i,t}\}}{e_{i,t}},$$

where *γ*_*u*_ and *γ*_*v*_ are parameters that determine the speed of adjustment and are in the interval [0, 1]. The *α*’s are probabilities and must satisfy 0 ≤ *α*_*u*,*i*,*t*_ ≤ 1 and 0 ≤ *α*_*v*,*i*,*t*_ ≤ 1.^2^ For the purposes of this article, we assume the adjustment speed for separations and vacancies are equal, i.e. *γ*_*u*_ = *γ*_*v*_ = *γ*.

Since the spontaneous and state-dependent processes are independent, the probability that a worker in occupation *i* is *not* separated from her job is (1 − *δ*_*u*_)(1 − *α*_*u*,*i*,*t*_). This means that the probability that a worker *is* separated is given by

2.10 $$\pi_{u,i,t}=1-(1-\delta_{u})(1-\alpha_{u,i,t})=\delta_{u}+\alpha_{u,i,t}-\delta_{u}\alpha_{u,i,t},$$

where the negative term on the right-hand side avoids counting a worker as separated twice. Similarly, for each employed worker in occupation *i*, the probability that a vacancy opens is

2.11 $$\pi_{v,i,t}=\delta_{v}+\alpha_{v,i,t}-\delta_{v}\alpha_{v,i,t}.$$

#### 2.2.3. Automation shocks

We assume that automation reallocates labour demand across occupations, decreasing the number of jobs available in some professions and increasing them in others. Since the set of occupations is fixed, we base the creation of new jobs on the thought experiment that work hours are reduced in all non-automated jobs, so that the total number of jobs in the economy stays constant. This assumption is motivated by the long-run evidence that unemployment rates have no trend but hours worked have decreased substantially [30]. In other words, we assume that aggregate labour demand remains constant during the shocks (i.e. $D_{t}^{†}=D_{0}=L$); automation reduces the target demand for occupations with a high automation level and correspondingly increases the target demand for occupations with a lower automation level, so that the number of jobs destroyed equals the number of jobs created (see Methods section S1.2 in the electronic supplementary material). As we discuss later, our model can also consider changes in the aggregate demand.

This completes our specification of the model. Table S1 of the electronic supplementary material gives a summary of the variables and parameters. For a full description of how we calibrated parameters and set initial conditions, see the Methods section S1.3 in the electronic supplementary material. Table S2 contains fitted values for all the parameters.

## 3. Results

### 3.1. Deterministic approximation for large populations

Although the workers and employers follow simple rules in our model, when the number of workers *L* is large, running the computer simulation is computationally costly. However, when *L* is large, we can take advantage of the law of large numbers and multivariate Taylor expansions to approximate the system’s behaviour in terms of expected values. This provides a good approximation for most purposes and makes it easier to understand the mechanics of the model and is faster to simulate, which is very useful for exploring the parameter space. We discuss these approximations in further detail in section S2 of the electronic supplementary material.

We approximate expectations for equations (2.2)–(2.4) in the limit of a large number of agents and conditional on the state of the system at the previous time step. To keep the notation compact, we often denote approximations to the expected values by a bar above the variable, e.g.

$${\bar{u}}_{i,t+1}\approx E[u_{i,t+1}|u_{i,t}={\hat{u}}_{i,t},v_{i,t}={\hat{v}}_{i,t},e_{i,t}={\hat{e}}_{i,t}].$$

We reduce the master equations to a 3*n* dimensional deterministic dynamical system given by

3.1 $$\begin{array}{rl} {\bar{e}}_{i,t+1} & ={\bar{e}}_{i,t}-\;\underset{\mathrm{separated}\;\mathrm{workers}}{\underbrace{(\delta_{u}{\bar{e}}_{i,t}+(1-\delta_{u})\gamma_{u}\max\{0,{\bar{d}}_{i,t}-d_{i,t}^{†}\})}}\\ & \;+\underset{\mathrm{hired}\;\mathrm{workers}}{\underbrace{\sum_{j}{\bar{\;f}}_{\;ji,t+1}}}, \end{array}$$

3.2 $$\;\begin{array}{rl} {\bar{u}}_{i,t+1} & ={\bar{u}}_{i,t}+\underset{\mathrm{separated}\;\mathrm{workers}}{\underbrace{(\delta_{u}{\bar{e}}_{i,t}+(1-\delta_{u})\gamma_{u}\max\{0,{\bar{d}}_{i,t}-d_{i,t}^{†}\})}}\\ & \;-\underset{\mathrm{transitioning}\;\mathrm{workers}}{\underbrace{\sum_{j}{\bar{\;f}}_{ij,t+1}}}\;, \end{array}$$

and

3.3 $$\begin{array}{rl} {\bar{v}}_{i,t+1} & ={\bar{v}}_{i,t}+\underset{\mathrm{opened}\;\mathrm{vacancies}}{\underbrace{(\delta_{v}{\bar{e}}_{i,t}+(1-\delta_{v})\gamma_{v}\max\{0,d_{i,t}^{†}-{\bar{d}}_{i,t}\})}}\\ & \;-\underset{\mathrm{hired}\;\mathrm{workers}}{\underbrace{\sum_{j}{\bar{\;f}}_{\;ji,t+1}}}. \end{array}$$

As we discuss in section S2.1 of the electronic supplementary material, we can express ${\bar{\;f}}_{ij,t+1}$ in terms of the adjacency matrix and the expected values of the state variables as follows:

3.4 $${\bar{\;f}}_{ij,t+1}=\frac{{\bar{u}}_{i,t}{\bar{v}}_{\;j,t}^{2}A_{ij}(1-{\text{e}}^{-{\bar{s}}_{\;j,t+1}/{\bar{v}}_{\;j,t}})}{{\bar{s}}_{\;j,t+1}\sum_{k}{\bar{v}}_{k,t}A_{ik}},$$

where

3.5 $${\bar{s}}_{\;j,t+1}=\sum_{i}\frac{{\bar{u}}_{i,t}{\bar{v}}_{\;j,t}A_{ij}}{\sum_{k}{\bar{v}}_{k,t}A_{ik}}$$

is the expected number of applications submitted to vacancies of occupation *j*. In section S2.1 of the electronic supplementary material, we discuss the relative error of this approximation and provide mathematical arguments that suggest that this error is inversely proportional to the number of agents. We run simulations to show that the error is negligible in the limit of a large number of agents (see section S2.2 of the electronic supplementary material). Therefore, we can use equations (3.1)–(3.3) to study large systems, in this case, the US labour market, in a tractable manner. We also use equations (3.2) and (3.4) to compute long-term unemployment (see section S1.4 in the electronic supplementary material).

Given a set of time series for the target labour demand $d_{i,t}^{†}$ and a set of initial conditions, equations (3.1)–(3.3) determine the expected employment, unemployment, and vacancies as a function of time. Our results are based on the US occupational mobility network, so we think of our model as reflecting the US with free geographical mobility. In section S2.2 of the electronic supplementary material, we show that our deterministic approximation is valid for a labour pool of at least 1.4 million workers, so our model could potentially be applied at the regional level.

#### 3.1.1. Steady state

Before proceeding to analyse the US labour market under a changing demand for labour, we note that for all the cases we have studied, when the target labour demand is constant $d_{i}^{†}$, there exists a computable steady-state value for the expected number of employed and unemployed workers and vacancies in each occupation. Except for the simple case of a complete network, with *A*_*ij*_ = 1/*n*, we cannot derive a closed-form solution for occupational unemployment. Nonetheless, we have solved equations numerically to find a solution. In section S2.3 of the electronic supplementary material, we argue that the steady-state values depend on the network structure and the target labour demand. Thus, the network structure, and the distribution of labour demand across occupations, can substantially influence the steady-state unemployment at both the occupational and the aggregate levels.

### 3.2. The Beveridge curve

**Figure 3.**
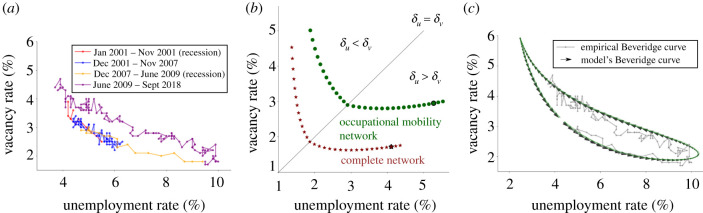
The Beveridge curve. In each panel, we plot the unemployment and vacancy rate. (*a*) The historical Beveridge curve for the United States, 2001–2018. Different periods are highlighted with different colours. (*b*) Movement of the Beveridge curve due to changes in labour market frictions. In particular, we plot the difference between a complete network, with no skill mismatch frictions versus the empirical occupational mobility network. Each dot corresponds to the steady-state unemployment and vacancy rate for for different pairs of values of *δ*_*u*_ and *δ*_*v*_. The highlighted points correspond to the unemployment and vacancy rate of the model using the calibrated parameters. (*c*) The Beveridge curve generated by our model. The parameters of the model are calibrated to match the empirical Beveridge curve between December 2007 and December 2018. The dashed lines correspond to the deterministic approximation of equations (3.1)–(3.3), and solid green lines to the full stochastic model simulation of equations (2.2)–(2.4). The transparent grey line shows the empirical Beveridge curve between December 2007 and September 2018.

The Beveridge curve is one of the best known macroeconomic stylized facts [20,21]. It states the relationship between vacancies and unemployment: when more vacancies open, unemployment goes down. The intuition is that when there are many vacancies, unemployed workers get a job faster, so the unemployment rate is low. Similarly, when there are few vacancies, unemployed workers are less likely to find jobs, so the unemployment rate is high. In figure 3*a*, we plot the Beveridge curve for the USA between January 2001 and September 2018.

The Beveridge curve has three important features: (i) the curve can shift away or towards the origin [31]. For example, after the 2009 financial crisis, the Beveridge curve shifted away from the origin, with unemployment increasing for all vacancy rates. (ii) During recessions, unemployment and vacancy rates move downwards along the curve, and during recovery periods, the unemployment and vacancy rates move upward along the curve. The recession from December 2007 to June 2009 and the recovery period from 2009 onwards are a good example of this feature (figure 3*a*). (iii) Historically, the Beveridge curve has (almost) always shifted outwards after recessions [21], i.e. the curve cycles anticlockwise. In other words, there are memory effects, and for the same vacancy rate, the unemployment rate has been larger during recoveries than during recessions. As we show later, our model reproduces these three features.

To estimate the impact of automation shocks, we need to calibrate the model with specific values for all the parameters. Given the importance of the Beveridge curve in the literature, we chose the parameters that maximize the ability of our model to reproduce it. To do so, we impose a simulated business cycle, i.e. we assume that the aggregate target labour demand *D*_*t*_ oscillates according to a sine wave. We then calibrate the amplitude of the sine wave and the parameters *δ*_*u*_, *δ*_*v*_ and *τ* to match the empirical Beveridge curve during the most recent US business cycle, from 2008 to 2018. We use the 2016 employment distribution across occupations to set the initial target labor demand across occupations (see calibration details in section S1.3 of the electronic supplementary material).

Our model reproduces the three mentioned features of the Beveridge curve. First, we show that structural changes, such as a decrease in worker–vacancy matching efficiency, cause the Beveridge curve to shift with respect to the origin. To demonstrate this, we hold the target aggregate demand *D*_*t*_ constant, and instead, vary the structure of the network by replacing the empirical network *A* with a complete network, where $A_{ij}=1/n\;\forall i,j$, in which each node is linked to every other node with equal weights. The complete network corresponds to the null hypothesis of no skill restrictions. We do this for different pairs of values of *δ*_*u*_ and *δ*_*v*_ and trace the *steady-state* behaviour in figure 3*b*. As expected, when we remove the network structure, the Beveridge curve shifts downwards towards the origin. When we consider parameters calibrated to actual data (highlighted with a bold border), removing the network structure corresponds to an increase in unemployment from 4.1% to 5.3%. This effect is substantial, representing a 29% increase.

Second, our model reproduces the dynamics of the Beveridge curve over business cycles. As shown in figure 3*c*, unemployment and vacancy rates move downwards along the curve during recessions and upwards along the curve during recovery periods.

Third, the Beveridge curve of our model cycles anticlockwise. Historically, the standard interpretation has been that these movements are shifts that correspond to structural changes (changes in parameters leading to a deterioration in the matching/hiring process in the economy) [21,32]. More recently, some models [23,29,33] have suggested that this phenomenon is independent of structural change and is instead due to the business cycle dynamics.

Our model supports this hypothesis. As shown in figure 3*c*, when we use the calibrated parameters, the Beveridge curve cycles in an anticlockwise direction even though we assume no structural changes. The fit of the Beveridge curve depends strongly on the parameters *δ*_*u*_ and *δ*_*v*_. In section S3 of the electronic supplementary material, we show how these parameters (as well as the network structure) can change the position, cycling direction and the area enclosed by the Beveridge curve. Yet, it is still possible that we are overfitting the Beveridge curve. To address these concerns, in section S4.2 of the electronic supplementary material, we explore an alternative calibration method for *δ*_*u*_ and *δ*_*v*_ that does not rely on the Beveridge curve. We show that although this new calibration affects the exact numbers of our estimates for the impact of automation on employment, the overall assessment for occupations and aggregate results remain robust. We also discuss alternative calibration procedures that could be sought in further work.

### 3.3. The impact of automation on employment

We now use the model to assess the impact of automation shocks on employment. We study two automation scenarios, one based on the study by Frey and Osborne [8] and the other based on the study of Brynjolfsson *et al*. [7]. We refer to these automation scenarios as the *Frey and Osborne shock* and as the *Brynjolfsson *et al.* shock*, respectively. For brevity, we show the figures for the Brynjolfsson *et al.* shock in section S4.3 of the electronic supplementary material.

#### 3.3.1. Estimates of the automation shock

Frey and Osborne estimated the probability that each of 702 occupations in the O*NET six-digit classification system could be computerized soon [8]. To do this, they gave experts a description of tasks performed by workers in a restricted sample of 70 occupations and asked them whether the occupations could be automated within the next two decades. Based on the experts’ answers and using nine O*NET variables that describe occupations as inputs, they trained a supervised machine learning algorithm and estimated what they called the *probability of computerization* for the remaining occupations. They found that approximately half of the jobs in the United States would be at risk of some degree of automation.

This study, as well as the Brynjolfsson *et al.* study (see electronic supplementary material, section S4.3), estimates the probability that an occupation will be *technically automatable*. This is not the probability that an occupation will be automated, which also depends on cost, institutions and so on, and it is not an estimate of the share of jobs in an occupation that will be automated. Nonetheless, for simplicity, we interpret these as automation levels, directly determining the share of jobs in an occupation that will be automated. We map the six- and eight-digit O-NET classifications used in these studies into the US occupational mobility network (which is based on the four-digit American Community Survey classification) using the 2016 National Employment Matrix Crosswalk (see reference [19]).

#### 3.3.2. Introducing automation shocks

**Figure 4.**
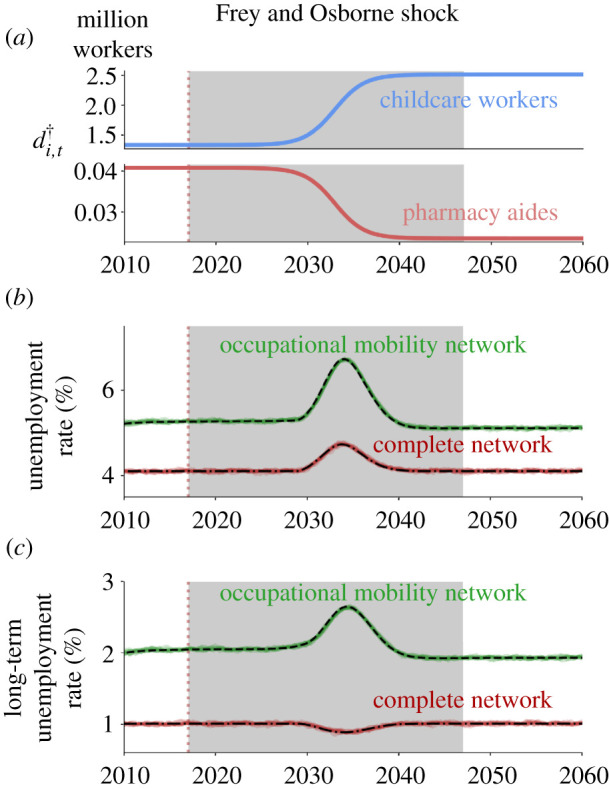
Aggregate labour market outcomes under the Frey and Osborne shock. The grey area denotes the 30 years during which the automation shock takes place. (*a*) The evolution of the target labour demand for two example occupations. The occupation coloured in blue has a low automation level and the occupation coloured in red has a high level. Because of its heterogeneity across occupations, the Frey and Osborne shock implies a large change in the target labour demand of most occupations. (*b*) The unemployment rate as a function of time. Dashed lines are our approximations of the expected value (solved numerically) and the solid lines are 10 simulations with 1.5 M agents. (*c*) The long-term unemployment rate as a function of time. As before, dashed lines correspond to the deterministic approximation of equations (3.1)–(3.3) and solid lines to the full stochastic model simulation of equations (2.2)–(2.4).

Before the automation shock, we assume the system is in a steady-state where the target demand $d_{i,0}^{†}$ matches the employment distribution in 2016 (see Methods section S1.3 in the electronic supplementary material). We then introduce an automation shock by making the target demand $d_{i,t}^{†}$ follow a sigmoid function, which begins at $d_{i,0}^{†}$ and converges to the post-automation target demand (see figure 4*a*). We choose the adoption rate so that the total shock is spread across a 30-year period, though most of the change happens within about 10 years. See Methods section S1.2 in the electronic supplementary material, for details. In section S4.4, we show that these results are fairly robust for reasonable adoption rates.

#### 3.3.3. Aggregate level outcomes

As shown in figure 4*b*,*c*, even though the aggregate target demand is held constant, the Frey and Osborne shock increases both the aggregate unemployment rate and the aggregate long-term unemployment rate during the period of automation. This increase is caused by the substantial reallocation of labour demand across occupations (see figure 4*a* for an example of how the target demand changes at the occupation level).

We compare the behaviour with the empirical occupational mobility network to the hypothetical behaviour assuming a complete network, in which any worker can transition equally well to any occupation. We use the same parameters for both networks (see calibrated parameter values in table S2 in the electronic supplementary material). The aggregate unemployment rate is initially about 5.3% for the empirical network and 4.1% for the complete network. When we apply the Frey and Osborne shock, the aggregate unemployment rate for the empirical network rises to 6.7% at its peak and then decays. In contrast, for the complete network, the aggregate unemployment rises to only 4.7% before it decays. Thus, the total change in unemployment with the empirical network is more than a factor of two larger, demonstrating the importance of the network structure.

The *long-term* unemployment rate, for the occupational mobility network, is about 2.1% for the empirical network, substantially smaller than the (short-term) unemployment rate. When we apply the Frey and Osborne shock, the aggregate long-term unemployment rate rises to roughly 2.6% at its peak and then decays. The relative change from the initial value to the peak value is about 27% for both the unemployment and long-term unemployment. The behaviour for the complete network is quite different: first, the initial level of long-term unemployment for the complete network is only 1.0%, more than a factor of two smaller than for the empirical network. Second, when we apply the shock, long-term unemployment for the complete network remains nearly flat.

Another interesting result is that the steady-state value of the aggregate unemployment shifts after the shock. The aggregate unemployment rate changes from 5.28% to 5.10%, for a net change of roughly −0.17%. While this is small, bear in mind that we have kept both the total aggregate target demand and all the model parameters constant. This is consistent with our result that the steady state explicitly depends on the network structure and the target demand in each occupation (see electronic supplementary material, eqs. (S60–S64) for details). The fact that we see this shift when we change the target demand demonstrates the key role that the network structure plays in determining the steady-state and transient behaviour. Note that there is no noticeable shift in the steady state for the complete network.

We conjecture that the Frey and Osborne shock causes such persistent effects since automation levels of neighbouring occupations tend to be similar. This has two effects: it means that there are some regions of the network where workers easily find new jobs, and others where workers get trapped because there are no good alternatives, causing a substantial boost to long-term unemployment. The steady-state shift occurs because the post-automation distribution of the target labour demand across occupations is more concentrated on fewer occupations that are more densely connected between each other, reducing worker–vacancy matching frictions. We test our conjecture by creating a surrogate Frey and Osborne shock that randomizes the distribution of automation levels of occupations across the network and find supporting results (see electronic supplementary material, section S5.1). Finally, we explore what happens when we relax the assumption that the aggregate target labour demand remains constant. Unsurprisingly, we find that when the aggregate demand decreases, the automation shock displaces more workers for all occupations (see section S4.5 in the electronic supplementary material).

#### 3.3.4. Occupation-level outcomes

We now show how automation affects the occupation-specific unemployment rates, where the network plays a crucial role. We measure the *average unemployment rate* and *average long-term unemployment rate* during the shock as follows:

$${\bar{u}}_{i,\mathrm{average}}(T)=\frac{\sum_{t\in T}{\bar{u}}_{i,t}}{\sum_{t\in T}({\bar{u}}_{i,t}+{\bar{e}}_{i,t})}$$

and

$${\bar{u}}_{i,\mathrm{average}}^{\left(\geq \tau \right)}(T)=\frac{\sum_{t\in T}{\bar{u}}_{i,t}^{\left(\geq \tau \right)}}{\sum_{t\in T}({\bar{u}}_{i,t}+{\bar{e}}_{i,t})}\;,$$

where *T* is the set of time steps that correspond to the automation shock. (We discuss an alternative way of defining the average unemployment rate in the electronic supplementary material, section S4.6 and show that our results are robust.) For simplicity, from here onward, we refer to the average unemployment rate and the average long-term unemployment rate during the automation period simply as the unemployment rate and the long-term unemployment rate.

**Figure 5.**
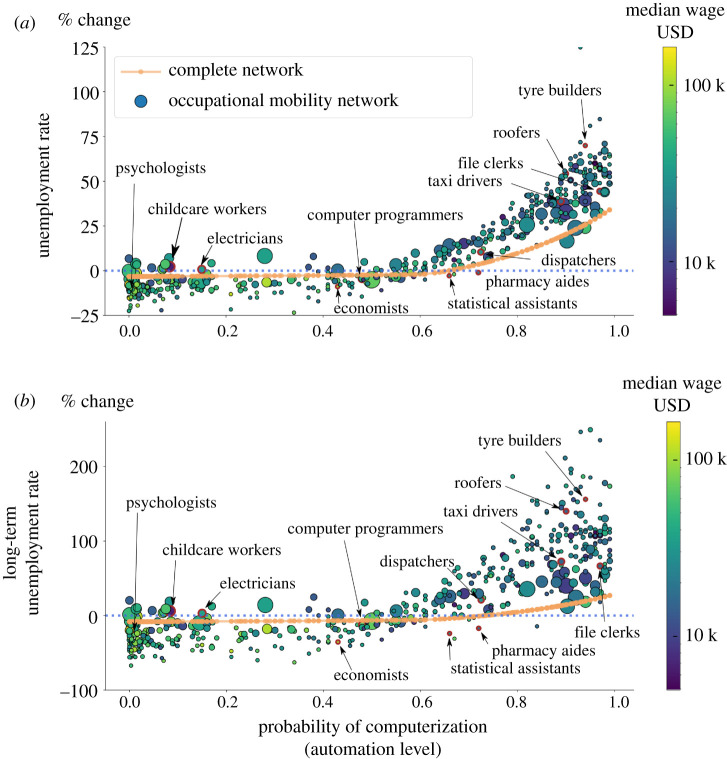
Impact of the Frey and Osborne shock on unemployment and long-term unemployment at the occupation level. The green dots are for the occupational mobility network, and the red dots are for the complete network. The size of the green dots is proportional to the employment of the occupation they represent. (*a*) The percentage change in the unemployment rate versus the automation level for each occupation, and (*b*) the same thing for the long-term unemployment rate. The scatter in the results demonstrates that, due to network effects, the automation level only partially explains occupational unemployment.

In figure 5, we compare the percentage changes in unemployment and long-term unemployment with each occupation’s automation level. To highlight the role of the network, we do this for both the occupational mobility network and the complete network. For the complete network, occupations with the same automation level have the same percentage change in their unemployment rates. In contrast, for the occupational mobility network, the automation level is *not* a perfect predictor of the occupation-level outcome. Network effects specific to each occupation also affect unemployment (this is also true for Brynjolfsson *et al.* [34] shock).

It is useful to highlight some specific cases to make the size of the network effects more clear. Both dispatchers and pharmacy aides have a high probability of computerization roughly of 0.72. Still the automation shock causes a 21% increase in the dispatchers’ long-term unemployment, while the pharmacy aides’ long-term unemployment *decreases* by roughly 17%. Some occupations experience the opposite change that one would expect. Statistical technicians and pharmacy aides are likely to be automated (with a probability of computerization above 0.6), while childcare workers and electricians are not (with a probability of computerization below 0.2). However, statistical technicians and pharmacy aides *decrease* their long-term unemployment, while childcare workers and electricians increase theirs. This is due to the fact that it is relatively easy for statistical technicians and pharmacy aides to transfer to jobs in other occupations with increasing demand. In contrast, it is easier for other workers with occupations susceptible to automation to transfer to childcare workers or electricians, thereby increasing workers’ supply relative to the demand. This illustrates the importance of network effects.

**Figure 6.**
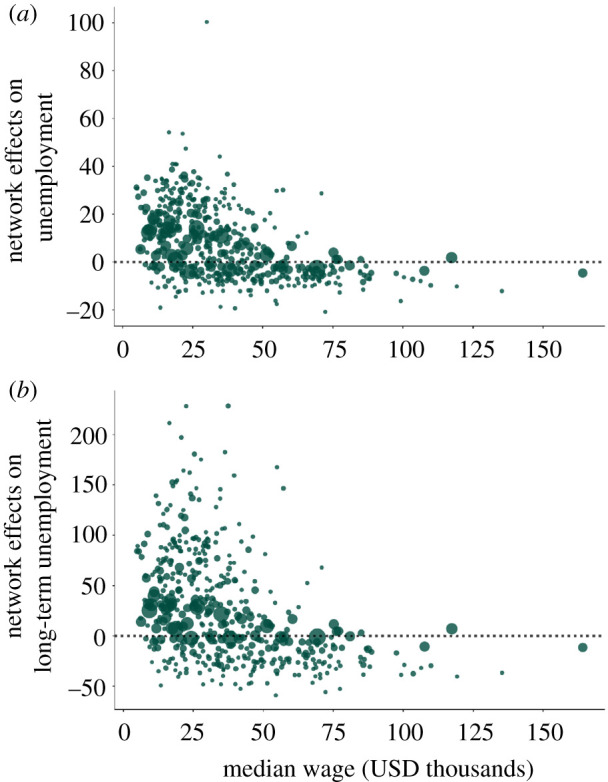
Network effects at the occupation level. We show the network effects for each occupation, i.e. the difference between the green and the black dots in figure 5. If this difference is positive, network effects are detrimental for the occupation. (*a*) Network effects on percentage change in unemployment rate versus median wage. (*b*) Network effects on percentage change in long-term unemployment rate versus median wage.

We also study if the network effects are beneficial or detrimental, that is, whether the occupation-specific unemployment rates decrease or increase when we consider the occupational mobility network instead of the complete network. We measure the network effects by taking the difference between the percentage change in unemployment rates in the two cases (i.e. the difference between the green and the black dots in figure 5). If the difference is positive, the network effects are detrimental—the occupation faces a larger increase in (long-term) unemployment. In figure 6, we compare the network effects with the median wage of occupations. We find that occupations with low median wage are more likely to face detrimental network effects, while almost all high wage occupations have better outcomes with the empirical network than with the complete network.

#### 3.3.5. Network structure

We further explore how the network structure affects the impact of automation on employment in the electronic supplementary material, section S5. First, we run the model on randomized occupational mobility networks (rewiring edges or reshuffling weights). Second, we model a retraining strategy by adding edges between occupations that share work activities and where one occupation substantially increases its long-term unemployment and the other decreases it. Our results show that adding these edges can help dampen the adverse effects of automation (see figure S26 in the electronic supplementary material).

#### 3.3.6. Brynjolfsson *et al.* automation shock

Brynjolfsson *et al.* [34] estimated the *suitability for machine learning* of occupations, which we use as an alternative automation shock (see electronic supplementary material, section S4.3 for details). Unlike the Frey and Osborne shock, the Brynjolfsson *et al*. shock causes no noticeable change in the aggregate unemployment rates. The different outcome of the model to the two automation shocks is caused by the different distributions of the two shocks. The Frey and Osborne shock is heterogeneous across occupations, affecting some occupations a great deal and others little, so that the changes in target demand at the occupation level are substantial (see figure 1*a*). In contrast, the Brynjolfsson *et al.* shock affects most occupations similarly, so that the changes in the target demand are lower and the network effects are small (see figure S15A in the electronic supplementary material). During the Brynjolfsson et al. shock, we still observe the network effects at the occupation level. The change in the long-term unemployment and unemployment varies substantially for occupations with similar suitability for machine learning (see figure S16A,B in the electronic supplementary material). However the network effects, workers of different occupations face during the Brynjolfsson *et al.* shock have no significant correlation with median wages (see figure S16C,D in the electronic supplementary material).

## 4. Discussion

This work develops an out-of-equilibrium model of the labour market and applies it to analyse the impact of automation on unemployment. At the occupation level, we show that employment impacts for workers are likely to depend not only on the automatability of their current occupation but also the alternative occupations that they can transition into. At the macro level, our model reproduces the dynamics of the Beveridge curve.

Similar to previous studies [11], we find that the occupational mobility network structure affects the unemployment rate. However, here we go further by quantifying the labour market frictions imposed by the empirical network: these frictions can account for more than a quarter of the steady-state unemployment rate. We also find that the distribution of labour demand across occupations in the network can affect the steady-state unemployment. Most importantly, automation can increase unemployment (and even more so long-term unemployment) during the transition period, even if the total number of jobs stays constant, due to the mismatch between unemployed workers and job vacancies. During this transition period, wages will play an important role. For the Frey and Osborne shock, our results suggest that low-wage occupations are more likely than high-wage occupations to see an increase in their long-term unemployment due to the frictions imposed by the occupational mobility network.

Our work complements previous efforts that have studied automation and job displacement based on the *task approach* [26,35,36], but provides a network perspective on job transitions that goes beyond classifying workers into low, middle and high skill categories. This article is also closely related to work that has used networks to study the effects of labour market frictions [37–39] or the propagation of economic shocks [12,24,40]. However, this study is the first to our knowledge to show that indirect network effects of occupational mobility can be crucial to estimate how automation may increase the unemployment and long-term unemployment of different occupations.

Our findings are particularly relevant for the macroeconomic literature on the Beveridge curve. Studies based on the search theory and networks have argued that structural changes can cause the shifts of the Beveridge curve [11,27,31,41]. Meanwhile, other studies suggest that these shifts are part of the Beveridge curve’s anticlockwise cyclicality, which results from business cycles dynamics [23,42]. While in our model, structural changes such as changes in the network structure do cause shifts in the Beveridge curve, our work supports the hypothesis that business cycles alone are enough to cause the Beveridge curve to cycle anticlockwise.

### 4.1. Policy implications

Some studies have focused exclusively on the automatability of occupations when assessing the outlook of workers. Here, we propose a wider view by considering not only the automatability of occupations but also workers’ possibilities for transitioning into occupations with open vacancies. In some cases, this perspective yields different and seemingly counter-intuitive results, where workers in some occupations at high risk of automation may actually have better employment prospects than workers is seemingly ‘safer’ occupations.

Our model can be particularly useful in helping policymakers target employment assistance packages and skill development programmes to workers who are more likely to face longer periods of unemployment. Our results suggest that there is a scope for retraining policies to leverage the occupational mobility network structure to reduce the adverse effects of automation. While this particular article has focused on labour market shocks relating to automation, our model is quite general and could also be adapted to analyse impacts arising from changes in labour demand relating to offshoring [43,44] or the transition towards the green economy [45].

## 5. Conclusion

We develop a data-driven, out-of-equilibrium model of the labour market that can be used to perform *in silico* experiments and has the potential to inform labour market policies. Our main result is that the network structure plays an important role in determining how automation affects unemployment. There is much scope for further work. Although our main result is robust to several parameter choices, the exact numbers of our estimates change with the parameters. To make accurate predictions, one would need to improve the calibration method and consider wage dynamics [31,41]. For example, one could attempt to calibrate the model using occupational level changes in employment due to previous recessions. We currently neglect wage dynamics to focus on labour market frictions due to worker–vacancy mismatches and because adding wages into the model requires vacancy data at the occupation level. While vacancy data are so far not publicly available, work is underway to prioritize data collection efforts to facilitate labour market research [46–48]. Finally, we have not considered the role of geography [12,49], cities [50] or the feedback effects from the production network [51], all of which are known to be important and would constitute crucial avenues for further research.

## Supplementary Material

Supplementary Material

## Endnotes

1 We use the empirical transition probability as a proxy for the relative preference with which a worker from occupation *i* applies to a job vacancy in *j*. We cannot use the actual probability with which a worker *applies* to a job vacancies of another occupation since this is not recorded in the census data. We discuss this further in section S1.1 of the electronic supplementary material.

2 Strictly speaking, one could impose a reallocation shock so large that $|d_{i,t}-d_{i,t}^{†}|>e_{i,t}/\gamma$ making one of the *α*s greater than one. This would only happen in exceptional circumstances and is very unlikely; nevertheless, in such a case, we simply set *α* = 1.

## Data accessibility

The code and data needed to reproduce the results of this paper are available at the online repository https://zenodo.org/record/4305783. The data used to construct the occupational mobility network was obtained from the Integrated Public Use Microdata Series, Current Population Survey [25].
