# Supplementary material for: Occupational mobility and automation: a data-driven network model
Source: J R Soc Interface. 2021 Jan 20;18(174):20200898. doi: 10.1098/rsif.2020.0898 (PMC7879770; doi:10.1098/rsif.2020.0898)
Supplement: Supplementary Material [file rsif20200898supp1.pdf]

Supplementary Material of
Occupational mobility and Automation: A data-driven network model

R. Maria del Rio-Chanona,[1,2*] Penny Mealy,[1,3,4,5] Mariano Beguerisse-Díaz,[2]
François Lafond,[1,2] and J. Doyne Farmer[1,2,6]

[1]Institute for New Economic Thinking at the Oxford Martin School, University of Oxford
[2]Mathematical Institute, University of Oxford
[3]Soda Laboratories, Monash Business School, Monash University
[4]School of Geography and Environment, University of Oxford
[5]Smith School of Environment and Enterprise, University of Oxford
[6]Santa Fe Institute,
[*]To whom correspondence should be addressed (rita.delriochanona@maths.ox.ac.uk).

Journal: Journal of the Royal Society Interface

Contents

S1 Methods **2**
- S1.1 Building the occupational mobility network . . . 3
- S1.2 Automation shocks . . . 4
- S1.3 Calibration . . . 6
- S1.4 Long-term unemployment . . . 8

S2 Mathematical derivations and approximations **10**
- S2.1 Deterministic approximation for large population . . . 10
  - S2.1.1 Separation and opening of vacancies. . . . 10
  - S2.1.2 Flow of workers approximation . . . 12
- S2.2 Simulations vs. approximation at the occupation level . . . 16
- S2.3 Fixed point and steady-state . . . 18
  - S2.3.1 Realized demand fixed-point equation . . . 18
  - S2.3.2 Employment, unemployment, and vacancies fixed-point equations . . . 19
  - S2.3.3 Complete network fixed-point . . . 20
  - S2.3.4 Zero steady-state . . . 22
  - S2.3.5 Long-term unemployment at the steady-state . . . 22

S3 The dynamics of the Beveridge curve 22

S4 Robustness 26
S4.1 Heterogeneous self-loops 26
S4.2 Parameter calibration robustness tests and validation discussion 27
S4.3 Brynjolfsson et al. shock 31
S4.4 Automation time and adoption rate 33
S4.5 Automation shocks that change aggregate labor demand 37
S4.6 A different measure of unemployment and long-term unemployment during automation 38

S5 The network structure, retraining effects, and steady-state shifts 41
S5.1 Shift in the steady-state unemployment post-automation 41
S5.2 Randomizing the network structure 42
S5.3 Retraining policies 45

S1 Methods

In this section, we first explain how we set the automation shocks and build the occupational mobility network. We then explain how we calibrate our model. Table S1 describes all variables and parameters needed to run the model, and Table S2 shows the calibrated values of the parameters.

**Main variables**	**Description**
$e_{i,t}$	Number of employed workers at time t in occupation i
$u_{i,t}$	Number of unemployed workers at time t who were last employed in occupation i
$v_{i,t}$	Number of job vacancies at time t of occupation i
**Other variables**	
$d_{i,t}$	Realized labor demand at time t of occupation i. ($d_{i,t} = e_{i,t} + v_{i,t}$)
$q_{ij,t}$	Probability that an unemployed worker from occupation i applies to a job vacancy of occupation j at time t
$p_{j,t}$	Probability that a job application sent to a vacancy of occupation j is successful
$s_{ij,t}$	Number of job applicants occupation j receives from workers of occupation i at time t
$s_{j,t}$	Number of job applicants occupation j receives at time t

$f_{ij,t}$	Flow of unemployed workers of occupation i becoming employed at occupation j
$u_{i,t}^{(k)}$	Number of unemployed workers of occupation i who at time t have already been unemployed for exactly k time steps
$\pi_{u,i,t}$	Occupation-specific probability that a worker of occupation i is separated
$\pi_{v,i,t}$	Occupation-specific probability that a vacancy opens in occupation i, per worker employed in occupation i
$\omega_{i,t}$	Number of workers from occupation i who are separated from their jobs at time t. Drawn from Binomial distribution $Bin(e_{i,t}, \pi_{u,i,t})$
$\nu_{i,t}$	Number of vacancies of occupation i opened at time t. Drawn from Binomial distribution $Bin(e_{i,t}, \pi_{v,i,t})$
**Parameters**	
δ_u	Rate at which employed workers are separated due to the spontaneous process
δ_v	Rate at which employed vacancies are opened due to spontaneous process
γ	Rate at employed workers and vacancies are separated or opened due to the market adjusting towards the target demand (state-dependent process).
τ	Number of time steps after which an unemployed worker is considered long-term unemployed
r	Weight of the self-loops of the occupational mobility network
A	Adjacency matrix of the occupational mobility network
$d_i^\dagger$	Post-automation target labor demand. Number of workers demanded at occupation i after the automation shock is complete
$d_{i,t}^\dagger$	Target labor demand of occupation i at time t
Δt	Duration of a time step in units of weeks

Table S1: Variables and parameters

S1.1 Building the occupational mobility network

Following Mealy et al. [8], we construct the occupational mobility network using empirical data on occupational transitions [3]. The classification is based on the 4-digit occupation codes,

which yields 464 distinct occupations. We used monthly panel data from the US Current Population Survey (CPS) to count the number of workers T_{ij} who transitioned from occupation i to occupation j during the period from January 2010 to January 2017. Letting $T_i = \sum_j T_{ij}$, we assume that if a worker changes occupation, the probability of transitioning from occupation i to occupation j as follows

$$P_{ij} = \frac{T_{ij}}{T_i}. \tag{S17}$$

We assume that the probability that a worker who changes jobs remains in the same occupation is constant across occupations for simplicity. Letting r be the probability that a worker who changes jobs stays in the same occupation, we write the adjacency matrix of the occupational mobility network as

$$A_{ij} = \begin{cases} r & \text{if } i = j, \\ (1-r)P_{ij} & \text{if } i \neq j. \end{cases} \tag{S18}$$

We estimate r based on the annual occupational mobility rate, which is the percentage of workers that switch occupations within a year [6]. Specifically, we calibrate r to roughly match the number of workers that annually change occupations in a year in the model with the empirical data (see Section S1.3)

While the empirical mobility network allows us to calibrate an occupational mobility network for us to use in our model, there are two different concepts to distinguish: The relative preference with which a worker from occupation i applies to a job vacancy in j (model) is different from the probability that a worker from occupation i, who is switching jobs, transitions to occupation j (empirical). However, the former is not directly observable from data. To overcome this issue, we use the occupational mobility network as indicative of the preference with which a worker from occupation i applies to a job vacancy in j. A caveat is that since the odds of a worker being hired do not uniquely depend on the preference with which workers apply to job vacancies, the transitions of workers observed in our model do not perfectly match the empirically observed transitions. Though the matching between the transitions in our model and the empirical network is not perfect, they are significantly similar – the Pearson correlation between them is 0.97.

S1.2 Automation shocks

Labor reallocation due to labor automation While it is clear that automation will replace some workers, this is an old process that has so far not caused persistent large unemployment rates [4]. Instead, the work week's average lengthwork week's average length has declined substantially [9] and work has shifted to new occupations [7]. Thus, we assume that automation will lead to a post-shock reallocation of labor demand, with some occupations increasing and others decreasing their labor demand.

Our model requires us to specify the post-shock reallocated demand $\mathbf{d}^\dagger$, which is the value to which the target labor demand converges after the shock. As we explain here, we use the probability of computerization scores of Frey and Osborne [5], and the suitability for machine learning scores of Brynjolfsson et al. [2] to set the post-shock reallocated demand. We denote by t^* the time at which the target demand converges to the post-shock reallocation demand.

First, we set the automation level of each occupation, which is bounded by 0 and 1, equal to the computerization probability or to the normalized suitability for machine learning scores[3] depending on the shock.

We assume that the automation level is the fraction of total hours worked in an occupation that are no longer needed post-shock. Furthermore, working hours are reduced for all workers in the economy, so that the total number of jobs stays constant. We denote the labor force, which is the number of workers, by L and assume that it remains constant. Let x_0 be the current number of labor hours for the average worker in a given period (say a week). The hours of work each occupation demands are given by the components of the vector

$$\mathbf{h}_0 = x_0 \mathbf{e}_0.$$

Letting $\mathbf{p}$ be the vector with the automation level of each occupation, the new number of hours of work $\mathbf{h}_{t^*}$ after automation is

$$\mathbf{h}_{t^*} = \mathbf{h}_0 \odot (\mathbf{1} - \mathbf{p}),$$

where $\odot$ denotes the element-wise multiplication of vectors and $\mathbf{1}$ the vector of ones. We split the aggregate hours of work equally among workers, thus the number of hours of work per week is

$$x_{t^*} = \frac{\sum_i^n h_{i,t^*}}{L}.$$

Finally, so that automation has no impact on the aggregate labor demand unemployment, we split the hours of labor demanded by occupations equally among workers,

$$\mathbf{d}^\dagger_{t^*} \equiv \mathbf{d}^\dagger = \mathbf{h}_{t^*} \frac{1}{x_{t^*}}. \tag{S19}$$

Of course, it is possible to assume that, rather than keeping the total number of jobs constant, automation either increases or decreases the total demand for jobs. We explore this in section S4.5.

Formulating a time dependent automation shock We follow the innovation literature, which suggests that the adoption of technologies follows a sigmoid function or S-curve over time [11]. Frey and Osborne say that their estimates are over "some unspecified number of years, perhaps

[3] We divide the score by 5, which is the maximum possible score, to normalize the scores between 0 and 1

a decade or two." [5]. Our baseline assumption is that the overall automation process happens within 30 years, but the steepest change within 10 years. We also explore different alternatives (see section S4.4).

We assume that the aggregate target demand equals the US labor force (i.e., $D^\dagger = L$). Initially, the target demand distribution equals the empirical employment distribution of occupations in 2016. At the end of the automation period, we assume that the target demand reaches the post-shock reallocated demand $\mathbf{d}^\dagger$. Within 15 years, the target demand is at the mid-point between the initial steady-state demand (given by the 2016 employment distribution) and the post-shock reallocated demand. We use a sigmoid function for the target demand

$$d^\dagger_{i,t} = \begin{cases} d_{i,0} & \text{if } t < t_s \\ d_{i,0} + \frac{d^\dagger_i - d_{i,0}}{1+e^{-k(t-t_0)}} & \text{if } t \geq t_s. \end{cases} \tag{S20}$$

where t_s is the time at which the automation shock starts and t_0 is 15 years after t_s. We use $k = 0.797$, which guarantees that the target demand equals the post-shock reallocate demand up to a 0.0001 tolerance.

Before introducing the automation shock, we first initialize the model so that it converges to the steady-state unemployment rate. After it reaches the steady-state, we introduce the target demand $d^\dagger_{i,t}$ as explained above. In Section S4.5, we demonstrate the robustness of the results under variations in the time span of the automation shock.

S1.3 Calibration

To calibrate the model we use fine-grained data when possible and aggregate data when this is not possible.

Initializing target labor demand. To calibrate the target labor demand when the shock begins, we assume that the labor market is initially in steady state, so that the distribution of target labor demand across occupation is equal to the distribution of employment in that occupation in 2016. We assume that the aggregate labor demand is constant through time.

Fitting δ_u and δ_v (spontaneous shocks), and Δt (time step duration). We simulate an idealized business cycle and adjust these three parameters to find the best match to the empirical U.S. Beveridge curve from December 2007 to December 2018. To create the artificial business cycle, we assume the aggregate target demand D_t follows a sine wave of the form $D_t = D_0 + a\sin(t/2\pi T_{\text{cycle}})$, where D_0 is the initial demand, and T_{cycle} is the period of the business cycle. Based on visual inspection, we assume that the empirical curve has traversed about three-quarters of a business cycle between December 2007 and December 2018. Thus

December 2007 is about a quarter of a cycle past the previous peak, and December 2018 is the new peak. This gives a period of the oscillation $T_{\text{cycle}} = 14.6$ years. (The assumptions about phase do not influence the fit, they only explain our reasoning in choosing T_{cycle}).

To match the time step duration Δt in our model with real-time units, we use the definition of long-term unemployment. The U.S. Bureau of Labor Statistics defines long-term unemployed workers as those who have an unemployment spell of 27 or more weeks[4]. Since τ is the number of time steps after which a worker is considered long-term unemployed, Δt satisfies that $\Delta t = \frac{27\text{weeks}}{\tau}$.

We assume the model is at its steady-state at the beginning of the simulation, with the initial target demand $d_0^\dagger$ of each occupation matching employment in 2016 (which is the most recent year where we have data for individual occupations). We then let the target demand $d_{i,t}^\dagger$ of individual occupations move in tandem according to the sine wave, so that each occupation makes a pro-rata change tracking D_t, i.e. $d_t^\dagger = d_0^\dagger + a\sin(t/2\pi T)$ and simulate the model.

We run an exhaustive search over possible values of the amplitude a of the sine function (which determines the business cycle's amplitude) and the parameters δ_u, δ_v, and τ. Once we determine τ we can calculate the time step duration using $\Delta t = \frac{27\text{weeks}}{\tau}$ and match the business cycle period T_{cycle}. The objective of the search is to minimize the discrepancy between the model and the empirical Beveridge curve. As a criterion for the goodness of fit, we compare the intersection of the enclosed areas. The objective function is

$$\min_{a,\delta_u,\delta_v,\tau} \frac{A_m \cap A_e}{A_m \cup A_e}, \tag{S21}$$

where A_m is the area enclosed by the Beveridge curve of the model, A_e is the area enclosed by the empirical Beveridge curve, $A_m \cap A_e$ is the intersection of their areas, and $A_m \cup A_e$ is the union of their areas. This method requires each Beveridge curve to enclose an area. The model's Beveridge curve is closed since the $sine$ wave is periodic. We close the empirical Beveridge curve by connecting the starting and endpoints. The optimal parameters are $a = 0.065$, $\tau = 4$ (i.e., $\Delta t = 6.75$ weeks), $\delta_u = 0.016$ and $\delta_v = 0.012$. The model's optimal parameters are reasonably stable concerning the optimal choice $a = 0.065$. For example, when we increase a by 10%, Δt remains constant while δ_u and δ_v increase roughly by 6%, and when we decrease a by 10%, Δt and δ_u remain constant while δ_v increases by less than 5%.

Calibrating r, the probability that a worker changing jobs remains within the same occupation. We are handicapped by the fact that this is not directly recorded. Still, we can use data on the annual occupational mobility rate, which is the percentage of workers that change occupations within a year, to infer this indirectly. Previous studies estimated that 19% of workers in the USA changed occupations in a year, i.e., that 81% did not change occupations in a

[4]https://data.bls.gov/timeseries/LNS13008636

year. A more recent study shows that in the Danish economy, the annual occupational mobility rate is 20% [6]. Therefore, we assume that each year 81% of workers remain in their current occupation and use this to estimate r, using the following approach.

In the previous section, we explained that we use the empirical occupational transitions to incorporate the relative preference with which a worker from occupation i applies to a job vacancy in j (for $i \neq j$). Consistent with this approach (and acknowledging the same caveats), here we use the fact that every year 81% of workers remain in their current occupation to calibrate the preference r with which workers chose to apply to job vacancies in their current occupation.

For simplicity, we consider the following abstraction. We assume that the probability that a worker does not change occupation in one time step is time-invariant and constant across occupations. Then, we observe that in the model, only workers who are unemployed change occupation. Thus, the probability that a randomly chosen worker *does not* change occupation in one time step is the probability $1-u$ that she is employed plus the probability u that she is unemployed times the probability r that she does not change occupation, that is $((1-u)+ur)$. Then, the probability x that a worker does not change occupations in y time steps is $x = ((1-u)+ur)^y$. Solving for r implies

$$r = \frac{x^{1/y} + u - 1}{u}. \tag{S22}$$

With the baseline parameters our model makes roughly $y = 52/6.75 = 7.7$ time steps in one year. Assuming $x = 0.81$, $\delta_u = 0.016$ and $u = 0.06$ (the average U.S. unemployment rate since the year 2000) gives the estimate $r = 0.55$. For the alternative parameters we obtain $r = 0.42$.

Calibrating γ, which is the rate at which the realized demand adjusts towards the target demand. Unfortunately, we have no empirical data to calibrate this parameter, but as we demonstrate in section S4.2, the results of the model are fairly insensitive to γ across a wide range of reasonable parameters. We choose $\gamma = 10\delta_u$.

S1.4 Long-term unemployment

We can compute the number of long-term unemployed workers in each occupation using Eqs. (12 – 14) as follows. The expected number of workers with an unemployment spell of k steps for occupation i at time t is the expected number of workers with an unemployment spell of $k-1$ steps at the previous time step times the probability that a worker of occupation i is not hired. Thus the expected number of unemployed workers of occupation i with an unemployment spell

Table S2: Parameter values after calibration (rounded)

Parameter	Value	Description
δ_u	0.0160	Rate at which employed workers are separated due to the spontaneous process.
δ_v	0.0120	Rate at which employed vacancies are opened due to the spontaneous process.
γ	0.160	Speed at which the realized demand adjust towards the target demand by separating workers or opening vacancies.
Δt	6.75	Duration of a time step in units of weeks.
r	0.55	Probability that a worker stays in the same occupation
τ	4	Time steps after which a worker is considered long-term unemployed

of k time steps $u_{i,t+1}^{(k)}$ is given by the recursive equation

$$\bar{u}_{i,t+1}^{(k)} = \bar{u}_{i,t}^{(k-1)} \left(1 - \frac{\sum_j \bar{f}_{ji,t}}{\bar{u}_{i,t}}\right), \tag{S23}$$

with $\bar{u}_{i,1}^{(1)} = \bar{\omega}_{i,1} = \bar{e}_{i,0}\pi_{u,i,t}$.

We use the The U.S. Bureau of Labor Statistics definition of long-term unemployment, where workers are considered long-term unemployed if they have an unemployment spell of 27 or more weeks[5]. Similarly, in our model the long-term unemployed workers are those who have been unemployed for τ or more time steps. Using Eq. (S23), we compute the expected number of long-term unemployed workers ($\bar{u}_{i,t+1}^{(\geq\tau)}$) by summing over all workers with an unemployment spell of τ or more time steps

$$\bar{u}_{i,t+1}^{(\geq\tau)} = \sum_{k=\tau}^{\infty} \bar{u}_{i,t+1}^{(k)}. \tag{S24}$$

[5]https://data.bls.gov/timeseries/LNS13008636

S2 Mathematical derivations and approximations

S2.1 Deterministic approximation for large population

Running the agent-based model is computationally costly. To calibrate the model, we have to run the model many times with different parameter choices. Therefore, it is useful to have analytical equations that approximate the agent-based model, which we can solve in seconds. In this section, we derive approximations for the agent-based model dynamics; these approximations are based on deriving expectations for the next time step, conditional on observing the current state of the system. A more in-depth mathematical analysis would need to show that the approximation can work iteratively between time steps. We do not derive a thorough mathematical proof or such mathematical analysis here, but we show that the approximations work well via simulations (see next subsection S2.2 and Fig. S7).

This section focuses on approximations within a time-step and condition calculations on the realization of the random variables at the previous time step (i.e., we have hats on all variables at time t denoting realized values).

We split this derivation into two. First, we discuss the approximation for the separation and opening of vacancies (the terms with the maximum functions) in Eqs. (12 – 14). We then discuss the approximation for the flow of workers.

S2.1.1 Separation and opening of vacancies.

In this subsection we show that in the limit of a large number of agents

$$\bar{\omega}_{i,t+1} = \delta \hat{e}_{i,t} + (1-\delta)\gamma \max\left\{0, \hat{d}_{i,t} - d^{\dagger}_{i,t}\right\} \approx E[\omega_{i,t+1} | e_{i,t} = \hat{e}_{i,t}, d_{i,t} = \hat{d}_{i,t}] \tag{S25}$$

and that

$$\bar{\nu}_{i,t+1} = \delta \hat{e}_{i,t} + (1-\delta)\gamma \max\left\{0, d^{\dagger}_{i,t} - \hat{d}_{i,t}\right\} \approx E[\nu_{i,t+1} | e_{i,t} = \hat{e}_{i,t}, d_{i,t} = \hat{d}_{i,t}]]. \tag{S26}$$

The separation and opening of vacancies depends on the difference between the realized demand $d_{i,t+1} = e_{i,t+1} + v_{i,t+1}$ and the target demand $d^{\dagger}_{i}$. It follows from Eqs. (2–4) that the realized demand is given by

$$d_{i,t+1} = \hat{d}_{i,t} - \omega_{i,t+1} + \nu_{i,t+1}. \tag{S27}$$

where $\omega_{i,t}$ and $\nu_{i,t}$ are binomial random variables of $\hat{e}_{i,t}$ draws and success probability $\pi_{u,i,t}$ and $\pi_{u,i,t}$ respectively. In the limit of a large number of agents we can approximate their distributions to Normal distributions, so that

$$\nu_{i,t+1} - \omega_{i,t+1} = \hat{e}_{i,t}(\pi_{v,i,t} - \pi_{u,i,t}) - \eta_{i,t+1} \tag{S28}$$

where

$$\eta_{i,t+1} \sim \mathcal{N}\left(0, \left(\pi_{u,i,t}(1-\pi_{u,i,t}) + \pi_{v,i,t}(1-\pi_{v,i,t})\right) e_{i,t}\right). \tag{S29}$$

Using Eqs. (10) and (11) we obtain

$$\begin{aligned} \hat{d}_{i,t+1} &= \hat{d}_{i,t} + \hat{e}_{i,t}\delta(1-\gamma)\left(\frac{\max\left\{0, d_i^{\dagger} - \hat{d}_{i,t}\right\}}{\hat{e}_{i,t}} - \frac{\max\left\{0, \hat{d}_{i,t} - d_t^{\dagger}\right\}}{\hat{e}_{i,t}}\right) + \eta_{i,t+1} \\ &= \hat{d}_{i,t} + \delta(1-\gamma)(d_i^{\dagger} - \hat{d}_{i,t}) + \eta_{i,t+1}. \end{aligned} \tag{S30}$$

In other words $d_{i,t}$ is normally distributed. This means that

$$E[\max\left\{0, d_i^{\dagger} - \hat{d}_{i,t}\right\}] = \max\left\{0, d_i^{\dagger} - \hat{d}_{i,t}\right\} + \epsilon \tag{S31}$$

where

$$\epsilon = \sqrt{\frac{2}{\pi}\left(\pi_{u,i,t}(1-\pi_{u,i,t}) + \pi_{v,i,t}(1-\pi_{v,i,t})\right) e_{i,t}}. \tag{S32}$$

To obtain the above equations we have used the fact that $\max\left\{0, b\right\} = \frac{b}{2} + \frac{|b|}{2}$ and that the expected value of the absolute value of a normally distributed function with variance σ^2 is $\sigma\sqrt{\frac{2}{\pi}}$. Similarly,

$$E[\max\left\{0, \hat{d}_{i,t} - d_i^{\dagger}\right\}] = \max\left\{0, \hat{d}_{i,t} - d_i^{\dagger}\right\} + \epsilon. \tag{S33}$$

It follows Eqs. (5 – 6), (8–11) and (S31–S33) that

$$E[\omega_{i,t+1}|e_{i,t} = \hat{e}_{i,t}, d_{i,t} = \hat{d}_{i,t}] = \delta\hat{e}_{i,t} + (1-\delta)\gamma\max\left\{0, \hat{d}_{i,t} - d_{i,t}^{\dagger}\right\} + (1-\delta)\gamma\epsilon \tag{S34}$$

and that

$$E[\nu_{i,t+1}|e_{i,t} = \hat{e}_{i,t}, d_{i,t} = \hat{d}_{i,t}] = \delta\hat{e}_{i,t} + (1-\delta)\gamma\max\left\{0, d_{i,t}^{\dagger} - \hat{d}_{i,t}\right\} + (1-\delta)\gamma\epsilon. \tag{S35}$$

The first two term scale linearly with the employment of occupations, while ϵ scales with the square root of the employment of occupations. This means that in the limit of a large number of agents we can make the approximation

$$E[\omega_{i,t+1}|e_{i,t} = \hat{e}_{i,t}, d_{i,t} = \hat{d}_{i,t}] \approx \bar{\omega}_{i,t+1} = \delta\hat{e}_{i,t} + (1-\delta)\gamma\max\left\{0, \hat{d}_{i,t} - d_{i,t}^{\dagger}\right\}$$

and that

$$E[\nu_{i,t+1}|e_{i,t} = \hat{e}_{i,t}, d_{i,t} = \hat{d}_{i,t}] \approx \bar{\nu}_{i,t+1} = \delta\hat{e}_{i,t} + (1-\delta)\gamma\max\left\{0, d_{i,t}^{\dagger} - \hat{d}_{i,t}\right\}.$$

Note that since we are interested in calculating unemployment, vacancy, and employment rates (i.e. we will divide by factor proportional to L) we are only interested in the terms that scale linearly with L.

S2.1.2 Flow of workers approximation

In this subsection we derive the following approximation for the flow of workers

$$\bar{f}_{ij,t+1} = \frac{\hat{u}_{i,t}\hat{v}_{j,t}^2 A_{ij}(1 - e^{-\bar{s}_{j,t+1}/\hat{v}_{j,t}})}{\bar{s}_{j,t+1}\sum_k \hat{v}_{k,t}A_{ik}} \approx E[f_{ij,t+1}|\mathbf{u}_{i,t} = \hat{\mathbf{u}}_{i,t}, \mathbf{v}_{i,t} = \hat{\mathbf{v}}_{i,t}, \mathbf{e}_{i,t} = \hat{\mathbf{e}}_{i,t}],$$

where

$$\bar{s}_{j,t+1} = \sum_i \frac{\hat{u}_{i,t}\hat{v}_{j,t}A_{ij}}{\sum_k \hat{v}_{k,t}A_{ik}}. \tag{S36}$$

We denote the number of workers from occupation i that apply to occupation j at time t by $s_{ij,t}$. Given that only unemployed workers apply for jobs and that $q_{ij,t}$ is the probability that a worker from occupation i applies to a vacancy of occupation j the expected number of applications submitted from occupation i to occupation j is

$$E[s_{ij,t+1}|u_{i,t} = \hat{u}_{i,t}] = \hat{u}_{i,t}q_{ij,t+1}. \tag{S37}$$

The labor flow $f_{ij,t+1}$ is equal to the number of workers from occupation i applying to occupation j, $\hat{s}_{ij,t+1}$, multiplied by the probability $p_{j,t+1}$ that each application is successful. (Once applications are sent to a vacancy, all applications have the same probability of being accepted, so p does not depend on i). The expected value is

$$E[f_{ij,t+1}|\mathbf{u}_{i,t} = \hat{\mathbf{u}}_{i,t}, \mathbf{v}_{i,t} = \hat{\mathbf{v}}_{i,t}, \mathbf{e}_{i,t} = \hat{\mathbf{e}}_{i,t}] = E\left[s_{ij,t+1}p_{j,t+1}|\mathbf{u}_{i,t} = \hat{\mathbf{u}}_{i,t}, \mathbf{v}_{i,t} = \hat{\mathbf{v}}_{i,t}, \mathbf{e}_{i,t} = \hat{\mathbf{e}}_{i,t}\right]. \tag{S38}$$

Letting the total number of applications $s_{j,t+1}$ to occupation j be

$$s_{j,t+1} = \sum_k s_{kj,t+1}, \tag{S39}$$

the fraction $p_{j,t+1}$ of successful applications is the ratio of the number of vacancies $m_{j,t+1}$ that successfully match to the total number of applications, i.e.

$$p_{j,t+1} = m_{j,t+1}/s_{j,t+1}. \tag{S40}$$

Matching. To simplify upcoming calculations that require derivatives, it is convenient to express the number of matches $m_{j,t+1}$ in terms of the exponential function. This is a standard approximation, and the derivation we present is based on [10]. Recall that $m_{j,t+1}$ is the number of vacancies that successfully match with a job applicant. Since employees hire a worker uniformly at random from the pool of applicants, then $m_{j,t+1}$ is equal to the number of job applications that receive at least one job application.

An unemployed worker who applies for a job in occupation j with $\hat{v}_{j,t}$ vacancies, will apply to a particular vacancy with probability $\frac{1}{\hat{v}_{j,t}}$. Thus the probability that the worker does *not* send her application to that vacancy is $1 - \frac{1}{\hat{v}_{j,t}}$. For $\hat{s}_{j,t+1}$ unemployed workers sending applications to occupation j, the probability that a particular vacancy does not receive an application is $(1 - \frac{1}{\hat{v}_{j,t}})^{\hat{s}_{j,t+1}}$. Since each vacancy receiving an application hires one worker, the expected number $\bar{m}_{j,t+1}$ of successful job applications is

$$\bar{m}_{j,t+1} = \hat{v}_{j,t} \left(1 - (1 - \frac{1}{\hat{v}_{j,t}})^{\hat{s}_{j,t+1}} \right).$$

Using the approximation that $(1 - \frac{1}{x})^y \approx e^{-y/x}$ for large x and y we obtain

$$\bar{m}_{j,t+1} = \hat{v}_{j,t}(1 - e^{-\hat{s}_{j,t+1}/\hat{v}_{j,t}}). \tag{S41}$$

Taylor approximation for flow of workers It follows from Eqs. (S38) and (S40) that the flow of workers depends on $s_{ij,t+1}$, $m_{j,t+1}$ and $s_{j,t+1}$. These variables are not independent, but here I show that in the large L limit We can approximate $E[f_{ij,t+1}|\mathbf{u}_{i,t} = \hat{\mathbf{u}}_{i,t}, \mathbf{v}_{i,t} = \hat{\mathbf{v}}_{i,t}, \mathbf{e}_{i,t} = \hat{\mathbf{e}}_{i,t}]$ by

$$\bar{f}_{ij,t+1} = \bar{s}_{ij,t+1}\hat{v}_{j,t}\frac{(1 - e^{-\bar{s}_{j,t+1}/\hat{v}_{j,t}})}{\bar{s}_{j,t+1}}. \tag{S42}$$

To derive this approximation it is useful to define

$$s_{j\backslash i,t+1} \equiv \sum_{k \neq i} s_{kj,t+1}, \tag{S43}$$

which is the number of applications occupation j receives from all unemployed workers except those from occupation i. Note that $s_{j,t+1} = s_{j\backslash i,t+1} + s_{ij,t+1}$. Using this fact, and Eq. (S38) we define the following multivariate form of the flow of workers

$$\begin{aligned} E[f_{ij,t+1}|\mathbf{u}_{i,t} = \hat{\mathbf{u}}_{i,t}, \mathbf{v}_{i,t} = \hat{\mathbf{v}}_{i,t}, \mathbf{e}_{i,t} = \hat{\mathbf{e}}_{i,t}] &= g(s_{ij,t+1}, s_{j\backslash i,t+1}) \\ &\equiv s_{ij,t+1}\hat{v}_{j,t}\frac{1 - e^{-\left(\frac{s_{ij,t+1}+s_{j\backslash i,t+1}}{\hat{v}_{j,t}}\right)}}{s_{ij,t+1} + s_{j\backslash i,t+1}}. \end{aligned} \tag{S44}$$

This definition will allow us to do a multivariate Taylor expansion of the function g around the expected value of $s_{ij,t+1}$ and $s_{j\backslash i,t+1}$).

Recall that, for fixed i, the random variables $s_{ij,t+1}$ follow a multinomial distribution with $\hat{u}_{i,t}$ trials and probabilities $q_{ij,t+1}$ for $j = 1, ..., n$. This means that $s_{ij,t+1}$ and $s_{il,t+1}$ are drawn from the same realization of the multinomial distribution, and are therefore correlated. However, $s_{ij,t+1}$ and $s_{kj,t+1}$ are drawn from different realizations (and distributions); thus they are independent.

In other words, the number of workers from occupation i that apply to occupation j is correlated with the number of workers from occupation i that apply to occupation l – if all $\hat{u}_{i,t}$ workers apply to occupation j it means that no workers from i applied to occupation l. However, since workers do not coordinate when sending applications, the fact that many or few workers from occupation i apply to occupation j says noting about the number of workers from occupation k that applied to occupation j. Of course, this is conditional on $\hat{v}_{j,t}$, the number of vacancies in occupation j at the previous time step.

It follows from the fact that $s_{ij,t+1}$ and $s_{kj,t+1}$ are independent and from Eq. (S43), that $s_{ij,t+1}$ is independent from $s_{j\setminus i,t+1}$. Furthermore, in the limit of a large number of agents, $\hat{u}_{i,t}$ is large, and the standard deviation of $s_{ij,t+1}$ is small in comparison to the average. The same is true for $s_{j\setminus i,t+1}$; we therefore expand $g(s_{ij,t+1}, s_{j\setminus i,t+1})$ in a Taylor series around the expected value of $s_{j,t+1}$ and $s_{j\setminus i,t+1}$ as follows,

$$\begin{aligned} \bar{g}(s_{ij,t+1}, s_{j\setminus i,t+1}) &= g(\bar{s}_{ij,t+1}, \bar{s}_{j\setminus i,t+1}) \\ &+ \frac{1}{2}\frac{\partial^2}{\partial s_{ij,t+1}^2}\big(g(\bar{s}_{ij,t+1}, \bar{s}_{j\setminus i,t+1})\big)\,\mathrm{Var}[s_{ij,t+1}] \\ &+ \frac{1}{2}\frac{\partial^2}{\partial s_{j\setminus i,t+1}^2}\big(h(\bar{s}_{ij,t+1}, \bar{s}_{j\setminus i,t+1})\big)\,\mathrm{Var}[s_{j\setminus i,t+1}] + \dots \end{aligned} \tag{S45}$$

Next, we now show that, in the limit of a large number of agents, the second and third terms are negligible in comparison to the first term. Exclusively for this derivation, we introduce the notation $v \equiv \hat{v}_{j,t}$, $x \equiv s_{ij,t+1}$ and $y \equiv s_{j\setminus i,t+1}$. we denote the expected value of a variable x by μ_x and the variance by σ_x^2, so that

$$\mu_x = \hat{u}_{i,t} q_{ij,t+1}, \tag{S46}$$

$$\mu_y = \sum_{k\neq i} \hat{u}_{k,t} q_{kj,t+1} \tag{S47}$$

$$\sigma_x^2 = \hat{u}_{i,t} q_{ij,t+1}(1 - q_{ij,t+1}), \tag{S48}$$

$$\sigma_y^2 = \sum_{k\neq i} \hat{u}_{k,t} q_{kj,t+1}(1 - q_{kj,t+1}), \tag{S49}$$

where we have used the formula for the expected value and variance of (independent) multinomial distributions. Note that μ_x, μ_y, σ_x^2, and σ_y^2 are proportional to the unemployment variables and therefore scale linearly with the number of agents L.

Using this notation and taking partial derivatives from Eq. (S44), we obtain

$$
\begin{aligned}
\bar{h}(x,y) &= h(\mu_x,\mu_y) + \sigma_x^2 \left[\left(\frac{v\mu_x}{(\mu_y+\mu_x)^3} - \frac{v}{(\mu_y+\mu_x)^2} \right) \left(1 - e^{-(\mu_x+\mu_y)/v}\right) \right. \\
&+ \left. \left(\frac{1}{\mu_y+\mu_x} - \frac{\mu_x}{(\mu_y+\mu_x)^2} \right) e^{-(\mu_x+\mu_y)/v} - \frac{1}{2} \frac{\mu_x}{v(\mu_y+\mu_x)} e^{-(\mu_x+\mu_y)/v} \right] \\
&+ \sigma_y^2 \left[\frac{v\mu_x(1-e^{-(\mu_x+\mu_y)/v})}{(\mu_x+\mu_y)^3} - \frac{\mu_x}{(\mu_x+\mu_y)^2} e^{-(\mu_x+\mu_y)/v} \right. \\
&- \left. \frac{1}{2} \frac{\mu_x}{v(\mu_y+\mu_x)} e^{-(\mu_x+\mu_y)/v} \right] + o(\frac{1}{L}),
\end{aligned}
\tag{S50}
$$

Since μ_x, μ_y, σ_x^2, σ_y^2 and v scale linearly with L, in the limit of a large number of agents these five variables are of the same order of magnitude. The higher moments terms in the expansion are at most of the order of $\frac{1}{L}$ given that the n-order partial derivatives are proportional to $\frac{1}{L^{n-1}}$. It follows from these observation and from Eqs. (S44) and (S50) that, in the limit of large number of agents, the first term of Eq. (S50) scales with L, while the other terms do not scale with L. In other words, in the limit of a large number of agents, we can approximate

$$
\bar{f}_{ij,t+1} = \bar{s}_{ij,t+1} \frac{\bar{v}_{j,t}^2 (1 - e^{-\bar{s}_{j,t+1}/\bar{v}_{j,t}})}{\bar{s}_{j,t+1}},
\tag{S51}
$$

where we have recovered our original notation. The relative error of this approximation is inversely proportional to the number of agents i.e., the relative error of our approximation is

$$
\left| \frac{E[f_{ij,t+1}|\mathbf{u}_t,\mathbf{v}_t;A] - \bar{f}_{ij,t+1}}{E[f_{ij,t+1}|\mathbf{u}_t,\mathbf{v}_t;A]} \right| \propto \frac{\kappa_{ij,t+1}}{L+\kappa_{ij,t+1}},
\tag{S52}
$$

where

$$
\begin{aligned}
\kappa_{ij,t+1} &= \sigma_x^2 \left[\left(\frac{v\mu_x}{(\mu_y+\mu_x)^3} - \frac{v}{(\mu_y+\mu_x)^2} \right) \left(1 - e^{-(\mu_x+\mu_y)/v}\right) \right. \\
&+ \left. \left(\frac{1}{\mu_y+\mu_x} - \frac{\mu_x}{(\mu_y+\mu_x)^2} \right) e^{-(\mu_x+\mu_y)/v} - \frac{1}{2} \frac{\mu_x}{v(\mu_y+\mu_x)} e^{-(\mu_x+\mu_y)/v} \right] \\
&+ \sigma_y^2 \left[\frac{v\mu_x(1-e^{-(\mu_x+\mu_y)/v})}{(\mu_x+\mu_y)^3} - \frac{\mu_x}{(\mu_x+\mu_y)^2} e^{-(\mu_x+\mu_y)/v} \right. \\
&- \left. \frac{1}{2} \frac{\mu_x}{v(\mu_y+\mu_x)} e^{-(\mu_x+\mu_y)/v} \right] + o(\frac{1}{L}).
\end{aligned}
\tag{S53}
$$

depends on the network structure (see section below for calculation of specific case), but does not scale with L. Therefore, when $L \to \infty$ the relative error is negligible.

Substituting Eqs. (7) and (S37) into Eq. (S51), we can write $\bar{f}_{ij,t+1}$ in terms of the adjacency matrix and the expected values of the state variables as

$$\bar{f}_{ij,t+1} = \frac{\hat{u}_{i,t}\hat{v}_{j,t}^2 A_{ij}(1 - e^{-\bar{s}_{j,t+1}/\hat{v}_{j,t}})}{\bar{s}_{j,t+1}\sum_k \hat{v}_{k,t}A_{ik}}, \tag{S54}$$

where

$$\bar{s}_{j,t+1} = \sum_i \frac{\hat{u}_{i,t}\hat{v}_{j,t}A_{ij}}{\sum_k \hat{v}_{k,t}A_{ik}}. \tag{S55}$$

We have derive our approximations within a time step, conditioning on the previous time step. In practice we do these approximations at each time step, i.e. we substitute $\hat{e}_{j,t}$ by $\bar{e}_{i,t}$, $\hat{u}_{j,t}$ by $\bar{u}_{i,t}$, and $\hat{v}_{j,t}$ by $\bar{v}_{i,t}$. As we show by simulations in section S2.2 these approximation works.

S2.2 Simulations vs. approximation at the occupation level

We show how our approximations compare with simulations at the occupation level. Additionally, we discuss how the automation shock impacts occupations differently. In particular, we focus on four occupations that we use as examples. For each, we compare the average of 10 simulations with our numerical solution. As shown in Fig. S7 our approximate solution closely matches the average for all occupations. Because of computational constraints, we simulate the model with roughly 1.4 million agents, which corresponds to one percent of the labor force (note that the larger the number of agents, the better the approximation).

We focus on four occupations, sales representatives, lawyers and judges, electricians, emergency management directors, whose empirical employment is $476,550$, 1.1 million, $632,965$, and 6267, respectively. Emergency management directors are among the occupations with the lowest employment, with only four others having less employment. We run the numerical simulations with a hundredth of the real labor force, so in our simulations, the target demand for each occupation is roughly $4,765$, $11,000$, $6,329$, and 63, respectively. As shown in Fig. S7 our approximations match the average unemployment and long-term unemployment rate of each occupation. Noticeably, the fluctuations are much larger for emergency management directors, which is the occupation with the smallest employment and target demand. When we run the simulations with 1.4 million, most occupations have a target demand consistently above 50. We can therefore conclude that our approximations work well for cities with a labor pool above 1.4 million.

The unemployment rate of different occupations follows different trajectories. Sales representatives, who are likely to be automated, have higher unemployment rates during the shock. Then, the unemployment rate returns to a steady-state with a value similar to before the shock. Instead, lawyers and judges, who are unlikely to be automated, have lower unemployment rates during the shock. However, after the shock, the steady-state unemployment is slightly higher

than it was before the shock. Finally, the electricians, who are unlikely to be automated, first decrease their unemployment rate and increase it during the automation shock. We explain this behavior as follows. During the first part of the automation shock, more electrician vacancies open, thus decreasing unemployment. Nevertheless, the automation shock also causes workers of nearby occupations to become unemployed. As the automation shock continues to separate workers of neighboring occupations, many of these unemployed workers apply for the electrician vacancies causing the electrician's unemployment rates to increase.

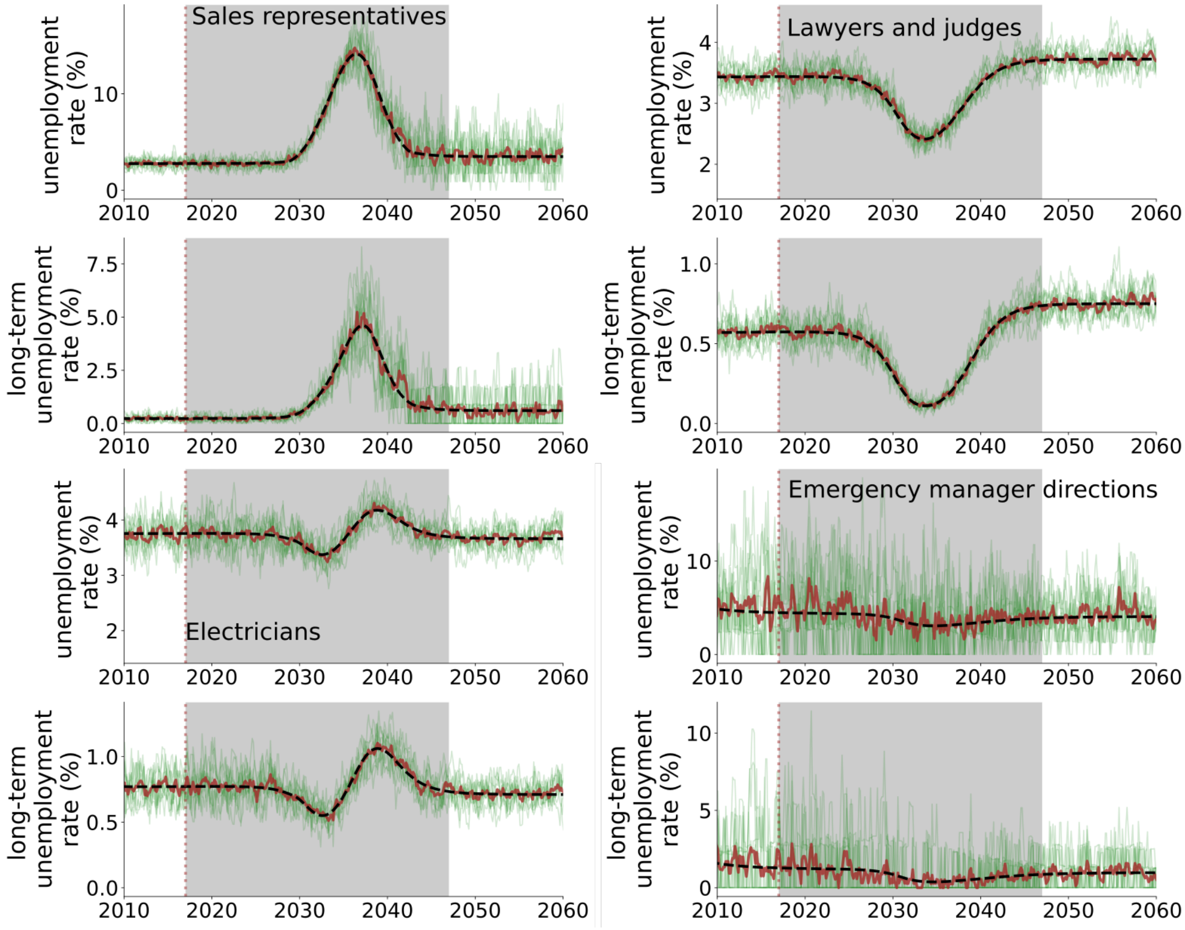

Figure S7: **Unemployment rate at the occupation level, simulations and numerical solution** We compare the unemployment and long-term unemployment from the average of the simulations (solid brown line) and the numerical solution (dashed line). We also show in transparent green lines the 10 simulations. Each simulation uses 1.4 Million agents and we average over 10 simulations.

S2.3 Fixed point and steady-state

In this section we derive fixed-point equations for the dynamical system given by Eqs. (12 – 14) under constant target demand, i.e., when $d^{\dagger}_{i,t} = d^{\dagger}_i \quad \forall i, t$. In other words, we find equations for employment, unemployment and vacancies whose solution $\mathbf{e}^*$, $\mathbf{v}^*$, and $\mathbf{u}^*$ satisfy that when $\bar{e}_{i,t} = \bar{e}^*_i$, $\bar{u}_{i,t} = \bar{u}^*_i$ and $\bar{v}_{i,t} = \bar{v}^*_i$, then Eqs. (12 – 14) imply $\bar{e}_{i,t+1} = \bar{e}_{i,t} = \bar{e}^*_i$, $\bar{u}_{i,t+1} = \bar{u}_{i,t} = \bar{u}^*_i$, and $\bar{v}_{i,t+1} = \bar{v}_{i,t} = \bar{v}^*_i$. These fixed-point equations depend on the parameters δ_u and δ_v, the network structure, and the target labor demand. We find an analytical solution to these fixed-point equations for a very simple case when the network is complete. When we consider the occupational mobility network, we can solve this equations numerically, as we have shown in the main paper (for example, see Fig. 3B). As we have shown by simulations, in the limit of a large number of agents, for all cases we studied, the steady-state corresponds to the solution of the fixed-point equations. Because of this, we use steady-state unemployment and fixed-point unemployment interchangeably in the main text.

We split this derivation into two, first we derive a fixed-point equation for the realized demand. We then show that this fixed point solution is consistent with a fixed point solution for employment, vacancies, and unemployment.

S2.3.1 Realized demand fixed-point equation

Since the realized demand is the sum of employment and vacancies, when we sum Eqs. (12 – 14) we obtain the following dynamic equation for the realized demand,

$$\bar{d}_{i,t+1} = \bar{d}_{i,t} + (\delta_v - \delta_u)\bar{e}_{i,t} + \begin{cases} \gamma_u(1-\delta_u)(d^{\dagger}_{i,t} - \bar{d}_{i,t}) & \text{if } \bar{d}_{i,t} \geq d^{\dagger}_{i,t} \\ \gamma_v(1-\delta_v)(d^{\dagger}_{i,t} - \bar{d}_{i,t}) & \text{if } \bar{d}_{i,t} < d^{\dagger}_{i,t}. \end{cases} \tag{S56}$$

We simplify this expression by defining $\gamma'_u = \gamma_u(1-\delta_u)$ and $\gamma'_v = \gamma_v(1-\delta_v)$ as follows,

$$\bar{d}_{i,t+1} = \bar{d}_{i,t} + (\delta_v - \delta_u)\bar{e}_{i,t} + \gamma'_u(d^{\dagger}_{i,t} - \bar{d}_{i,t}) + (\gamma'_v - \gamma'_u)\max\left\{0, d^{\dagger}_{i,t} - \bar{d}_{i,t}\right\}. \tag{S57}$$

Since the target demand is constant, when we apply the fixed-point condition $\bar{d}_{i,t+1} = \bar{d}_{i,t}$, we obtain the following equation

$$(\delta_u - \delta_v)\bar{e}^*_i = \gamma'_u(d^{\dagger}_i - d^*_i) + (\gamma'_u - \gamma'_v)\max\left\{0, d^{\dagger}_i - \bar{d}^*_i\right\}, \tag{S58}$$

where $\bar{e}^*_i$ is an employment fixed-point. Later on we derive and equation for e^*_i consistent with this equation. Let us consider the case $\delta_u > \delta_v$. To solve the above equation we consider the two possible which affect the maximum functions, $d^{\dagger}_i < d^*_i$ and $d^{\dagger}_i > d^*_i$. If $d^{\dagger}_i < d^*_i$, Eq. (S58) yields

$$d^{\dagger}_i - d_i* = \frac{\delta_u - \delta_v}{\gamma'_u}\bar{e}_{i,t}$$

which is a contradiction since the right hand side is positive, but the left hand side negative (since $\delta_u > \delta_v$). In the case when $d_i^\dagger > d_i^*$, we find the following fixed-point solution

$$d_i^* = d_i^\dagger - \frac{\delta_u - \delta_v}{\gamma_v'} \bar{e}_i^*.$$

Doing an analogous analysis for the case $\delta_u < \delta_v$ we obtain the following fixed-point solution for the realized demand,

$$\bar{d}_i^* = \begin{cases} d_i^\dagger - \frac{\delta_u - \delta_v}{\gamma_v'} \bar{e}_i^* & \text{if } \delta_u \geq \delta_v \\ d_i^\dagger + \frac{\delta_v - \delta_u}{\gamma_u'} \bar{e}_i^* & \text{if } \delta_u < \delta_v. \end{cases} \tag{S59}$$

In other words, when $\delta_u > \delta_v$ the realized demand is smaller than the target demand at a fixed-point. This happens because when $\delta_u > \delta_v$ (i.e., the probability of separation is greater than the probability of opening a vacancy at random) the adjustment towards the target demand does not fully compensate for asymmetry between the opening and separation rates; thus a fixed-point realized demand is smaller than the target demand. Similarly, when $\delta_u < \delta_v$, a fixed-point realized demand is greater than the target demand. In both cases, the difference between a fixed-point realized and the target demand is proportional to $|\delta_u - \delta_v|$ and inversely proportional to the adjustment rate γ. Except for the case when $\delta_u = \delta_v$ a fixed-point realized demand depends on $\bar{e}_i^*$.

S2.3.2 Employment, unemployment, and vacancies fixed-point equations

We use Eq. (S59) to derive a fixed-point equation for vacancies. We focus on the case $\delta_u \geq \delta_v$ since the other case can be solved analogous. It follows from Eq. S59) and the fact that the realized demand is the sum of employment and vacancies that a fixed-point solution for the vacancies must satisfy that

$$\bar{v}_i^* = d_i^\dagger - \left(1 - \frac{\delta_u - \delta_v}{\gamma_v'}\right) \bar{e}_i^*. \tag{S60}$$

When $\delta_u \geq \delta_v$, $d_i^\dagger < d_i^*$, so the maximum term is zero in the employment and unemployment equations (Eqs. (12) and (13)). It follows that, under the fixed point condition $\bar{e}_{i,t+1} = \bar{e}_{i,t}$, Eq. 12) leads to

$$\bar{e}_i^* = \frac{1}{\delta_u} \sum_j \bar{f}_{ji}^*, \tag{S61}$$

where $\bar{f}_{ji}^*$ is the flow of workers at the steady state and is given by

$$\bar{f}_{ij}^* = \frac{\bar{u}_i^* (\bar{v}_j^*)^2 A_{ij} (1 - e^{-\bar{s}_j^*/\bar{v}_j^*})}{\bar{s}_j^* \sum_k \bar{v}_k^* A_{ik}}, \tag{S62}$$

with

$$\bar{s}_j^* = \sum_i \frac{\bar{u}_i^* \bar{v}_j^* A_{ij}}{\sum_k \bar{v}_k^* A_{ik}}. \tag{S63}$$

Using Eq. (S61) and imposing the fixed-point condition $\bar{u}_{i,t+1} = \bar{u}_{i,t}$ we find that

$$\frac{1}{\delta_v} \sum_j \bar{f}_{ji}^* = \frac{1}{\delta_u} \sum_j \bar{f}_{ij}^*. \tag{S64}$$

In other words, at a fixed-point the total inflow of workers into an occupation is proportional to the total outflow of workers of that same occupation. When $\delta_u = \delta_v$ these two flows are equal.

Eqs. (S61) and (S64) are non-linear functions of $\mathbf{e}^*$, $\mathbf{u}^*$, and $\mathbf{v}^*$. We have not found a general closed-from solution for these equations, but we can make three important observations. i) Eq. (S60) shows that a fixed-point solution will depend on the target demand, which is an external input to the model. ii) Eq. (S61) shows that an employment fixed-point depends on the flow of workers between occupations, which depends on the network structure (see Eq. (15)). Therefore the network structure plays a role in determining the steady-state. iii) Eq. (S64) shows that a fixed-point solution will also depend on the parameters δ_u and δ_v. Finally, even though we do not have an analytical solution for Eqs. (S60) (S61), and (S64), we have found fixed-point solutions using computer calculations for all the networks and parameters we have studied. We have only found one non-trivial fixed-point solution for each case, i.e, only one solution besides the trivial solution $\mathbf{e}^* = \mathbf{v}^* = \mathbf{u}^* = \mathbf{0}$.

S2.3.3 Complete network fixed-point

Our model has an analytically computable fixed-point solution under the following assumptions: i) a complete network of n nodes, i.e., $A_{ij} = \frac{1}{n} \quad \forall i, j$, ii) $\delta_u = \delta_v = \delta$ and that $\gamma_u = \gamma_v = \gamma$, and iii) the target labor demand is constant, equal to the labor supply, and distributed homogenously among all occupations i.e., $\bar{d}_i^\dagger = \frac{L}{n} \quad \forall i$. As before, we denote a fixed-point value of the variables with a star superindex (e.g. x^*).

In the main text we show that the steady-state depends on the target demand and the network structure. Since, in this scenario, all occupations have equal target demand and are positioned indistinguishably in the network, all occupations have the same fixed-point. Therefore, we lose the i subindex in our notation. Since $\delta_u = \delta_v$, Eq. (S64) yields $\sum_j \bar{f}_{ji}^* = \sum_j \bar{f}_{ij}^* \equiv F^*$. Using the full expression for the flow of workers in Eq. (15) we obtain

$$F = \sum_{j=1}^{n} \frac{1}{n} \frac{u^* v^{*2}(1 - e^{s^*/v^*})}{s^* \sum_{k=1}^{n} \frac{1}{n} v^*} = \frac{u^* v^* (1 - e^{-s^*/v^*})}{s^*}.$$

Similarly, it follows from Eq. (S37) that

$$s^* = \sum_{i=1}^{n} \frac{1}{n} \frac{u^* v^*}{\sum_{k=1}^{n} \frac{1}{n} v^*} = u^*.$$

We then substitute s^* in F and obtain,

$$F = v^*(1 - e^{-\bar{u}^*/\bar{v}^*}) \tag{S65}$$

It follows from assumption ii) and Eq. (S59) that $\bar{d}^* = d^\dagger$. With this in mind, using Eq. (S65) and Eqs. (12)–(14), we obtain the following dynamic equations for the total number of employed and unemployed workers and job vacancies,

$$\bar{e}^* = \bar{e}^* - \delta\bar{e}^* + v^*(1 - e^{-u^*/v^*}) \tag{S66}$$

$$u^* = u^* + \delta\bar{e}^* - v^*(1 - e^{-u^*/v^*})$$

$$v^* = v^* + \delta\bar{e}^* - v^*(1 - e^{-u^*/v^*}).$$

We know that the number of unemployed and employed workers equals the labor, i.e., $U^* + E^* = L$. Since all occupations have the same steady state, then $\bar{u}^* + \bar{e}^* = \frac{L}{n}$. It follows from this observation, the fact that $d^* = \frac{L}{n}$, and Eq. (S59) that

$$\bar{u}^* = \frac{L}{n} - \bar{e}^* = \bar{v}^*. \tag{S67}$$

We then substitute Eq. (S67) into Eq. (S66) and obtain

$$\bar{e}^* = \frac{L}{n} \frac{(1 - e^{-1})}{\delta + (1 - e^{-1})}, \tag{S68}$$

and

$$u^* = v^* = \frac{L}{n} \frac{\delta}{\delta + (1 - e^{-1})}. \tag{S69}$$

These equations along with the condition of constant labor force imply that the unemployment rate is

$$\frac{U^*}{L} = \frac{\delta}{\delta + 1 - e^{-1}}. \tag{S70}$$

In other words, in this case the unemployment rate is an increasing function of δ.

S2.3.4 Zero steady-state

Eqs. (12) – (14) accept a trivial fixed-point $e_i^* = u_i^* = v_i^* = d_i^\dagger = 0$. We neglect this steady-state since it is uninteresting for our analysis. However, when running the agent simulation there is a non-zero probability that the number of employed workers and vacancies of an occupation is zero, i.e., $\hat{e}_{i,t} = \hat{v}_{i,t} = 0$. At this point, even if $d_{i,t}^\dagger > 0$, no vacancies would open and therefore employment would be zero for the rest of the simulation. To avoid this, we introduce the additional rule that if $\hat{e}_{i,t} = \hat{v}_{i,t} = 0$ but $d_{i,t}^\dagger > 0$, then a vacancy is opens. When running the simulation with a large number of agents (which is the case of the labor market) the probability that $\hat{e}_{i,t} = \hat{v}_{i,t} = 0$ is negligible and this additional rule is very unlikely to be used.

S2.3.5 Long-term unemployment at the steady-state

We note that Eq. (S24) gives the number of long-term unemployed workers for time t. A special case is that of the steady-state when the unemployment rate of each occupation is u_i^*. Then, the approximate expected number of unemployed workers with a job spell of k time steps is

$$\bar{u}_i^{*(k)} = \delta \bar{e}_i^* \left(1 - \frac{\sum_j \bar{f}_{ij}(\bar{\mathbf{u}}^*, \bar{\mathbf{v}}^*; A)}{\bar{u}_i^*}\right)^k.$$

and decays exponentially with k.

S3 The dynamics of the Beveridge curve

Undertaking a detailed assessment of the exact behavior of the Beveridge curve under different parameter choices is out of scope for this paper. However, we do briefly discuss how the direction in which the Beveridge curve cycles is influenced by the state-independent rates at which workers are separated (δ_u) and vacancies open (δ_v).

We explore the dynamics of the Beveridge curve by varying δ_u and δ_v. We keep all other parameters fixed to the values used to fit the Beveridge curve ($a = 0.065$, $\Delta t = 6.75$ weeks, and $\gamma_u = \gamma_v = \gamma = 0.16$). As before, we use a sine wave to model business cycle dynamics. In the top left of Figs.S8 and Fig.S9) we show the dynamics of the aggregate target demand. We start with a constant target demand and then introduce the business cycle dynamics. We show the first part of the dynamics with a dashed line to mark the transition between a constant target demand and an oscillating target demand. Then, we plot in color-scale the dynamics of a business cycle; the purple/blue part corresponds to the recession period, while the green/yellow part to the recovery period.

We test five different parameter options for δ_u and δ_v. Starting from the calibrated values $\delta_u = 0.016$ and $\delta_v = 0.012$, we gradually decrease δ_u by 0.001 and increase δ_v by 0.001 until

$\delta_u = 0.012$ and $\delta_v = 0.016$. This yields 5 different cases, which we show in Fig.S8 for the occupational mobility network and in Fig.S9 for the complete network.

We find that the Beveridge curve first reduces its enclosed area, then it changes its cycling direction from counter-clockwise to clockwise, and finally, it increases its enclosed area. For the two networks and for the five cases we study, we observe that when $\delta_u > \delta_v$, the curve cycles counter-clockwise. On the contrary, when $\delta_v > \delta_u$ the curve cycles clockwise. However, when $\delta_u = \delta_v$ the network determines the direction of the cycle – the occupational mobility network shows clockwise cycles while the complete network shows an "8"-shaped curve, where the bottom part cycles counter-clockwise and the upper part clockwise. These results suggest that for some similar values of δ_u and δ_v, which depend on the network structure, the curve flips and starts to exhibit the opposite cycling direction. We also observe that the network structure affects the area enclosed by and the position of the curve (see differences between Fig.S8 and Fig.S9).

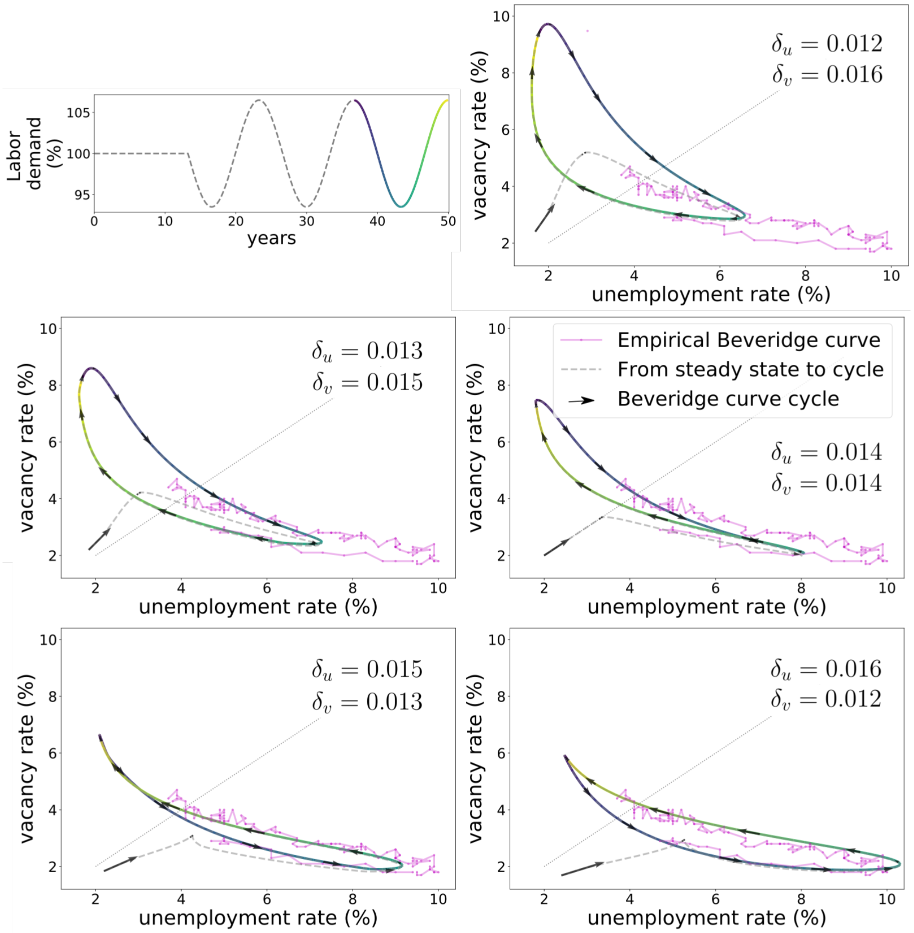

Figure S8: **Beveridge curve dynamics for the occupational mobility network.** On the top left panel we show the aggregate demand. The grey part corresponds to the steady-state and transition to the business cycle. The purple/blue part corresponds to the recession period, while the green/yellow part to the recovery period. The following 5 panels show the dynamics of the model's Beveridge curve under different parameter choices. We observe that when $\delta_u > \delta_v$ the curve cycles counter-clockwise, while when $\delta_u \leq \delta_v$ the curve cycles clockwise. We also show the empirical Beveridge curve in magenta for reference.

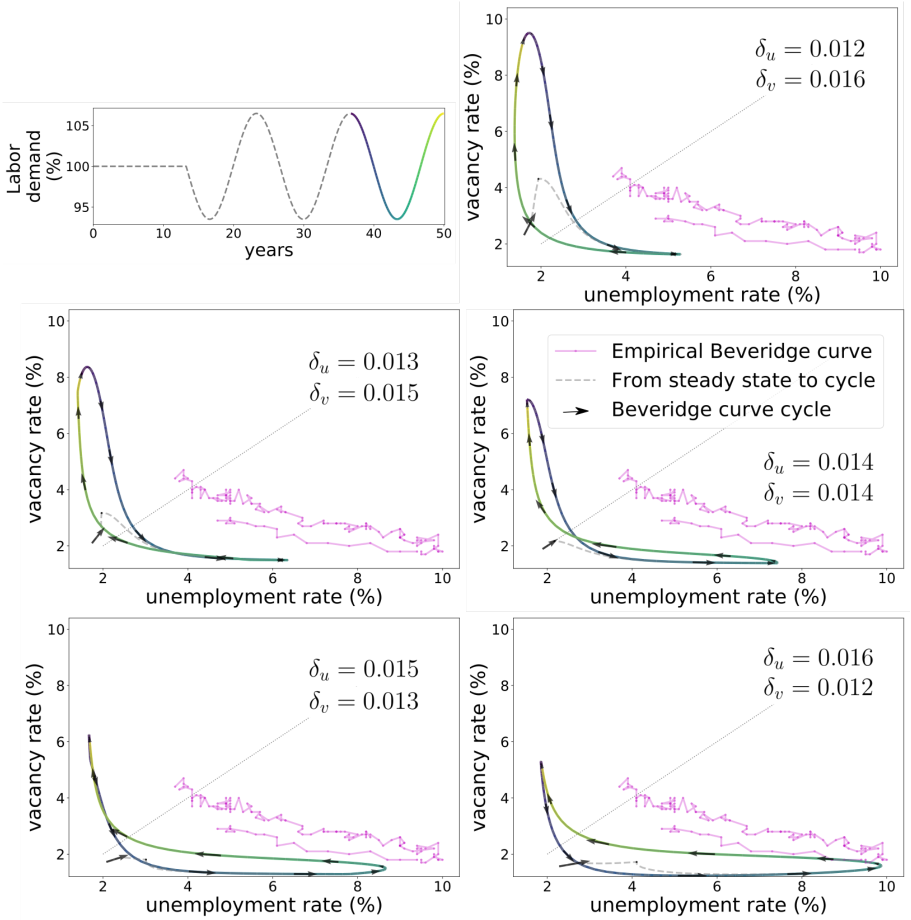

Figure S9: **Beveridge curve dynamics for the complete network** On the top left panel we show the aggregate demand. The grey part corresponds to the steady-state and transition to the business cycle. The purple/blue part corresponds to the recession period, while the green/yellow part to the recovery period. The following 5 panels show the dynamics of the model's Beveridge curve under different parameter choices. We observe that when $\delta_u \geq \delta_v$ the curve cycles counter-clockwise, while when $\delta_u < \delta_v$ the curve cycles clockwise. We also show the empirical Beveridge curve in magenta for reference.

S4 Robustness

In this section present our robustness analysis for the results.

S4.1 Heterogeneous self-loops

Due to a lack of quality data on workers that switch employers but stay in the same occupation, we assumed that the self-loops weight is the same for all occupations, $r_i = r \quad \forall i$. In this section, we explore a way of calibrating heterogeneous self-loops, that is, allowing each occupation i to have a self-loop with weight r_i.

While same-occupation job switches are scarcely recorded in the census, we can use some census information to define heterogenous self-loops after making some assumptions. IPUMS Current Population Survey has an 'EMPSAME' variable indicating whether a person's employer is still the same. However, this data has significant quality issues. Specifically, IPUMS reports that "there are a large number of people in all samples who appear to be in-universe for EMPSAME, that is, they are currently employed and were employed in the previous month, but still have NIU (Not in Universe) values for EMPSAME".

When we use these data to estimate the self-loops, we find unrealistically low values. For example, 41 occupations would have a zero self-loop, meaning that workers in these occupations always change occupation when they change employer. Furthermore, the highest self-loop weight we observe is 0.19, corresponding to lawyers and judges. This would imply that four-fifths of lawyers and judges change occupation when they change employer. However, even though the self-loop weights are unrealistically low, we find that the heterogeneity between occupations is reasonable. Licensed occupations such as lawyers and judges, nurses, and surgeons have high self-loops compared to the rest of the occupations. In the next paragraph, we explain how, with some assumptions, we can use this heterogeneity in the data of workers remaining or not in the same occupation to set a relative likelihood of workers to remain in their occupation.

First, we assume that occupations have positive self-loop weight i.e., $r_i > 0 \quad \forall i$. We assign a 0.0007 self-loop weight to the 41 occupations which previously had $r_i = 0$. We choose 0.0007 since it is the minimum positive weight of the original distribution of self-loops. Second, we define linearly re-scaled r'_i weights, which we obtain using the functional form $r'_i = mr_i + b$, where m and b are parameters and r_i is the self-loop weight of occupation i for the original distribution. We search for parameter values of m and b that satisfy the conditions that i) the maximum self-loop weight is 0.95 and ii) that $\frac{1}{n}\sum_{i=1}^{n} r_i = r$, where r is the homogenous self-loop weight we calibrated as explained in section S4.2. In other words, we add the condition that the mean of the distribution of the re-scaled self-loops should equal the weight of the homogenous self-loop we previously calibrated to match the annual occupational mobility rate. The parameter values we find are $m = 0.5$ and $b = 0.5$. We used the re-scaled self-loop weight

r_i' to run the model with heterogenous self-loops.

Our results vary little when we introduce heterogeneous self-loops. As we show in Fig. S11 the effect is mild even at the aggregate level. Few occupations deviate from the identity, and none differ by a large amount.

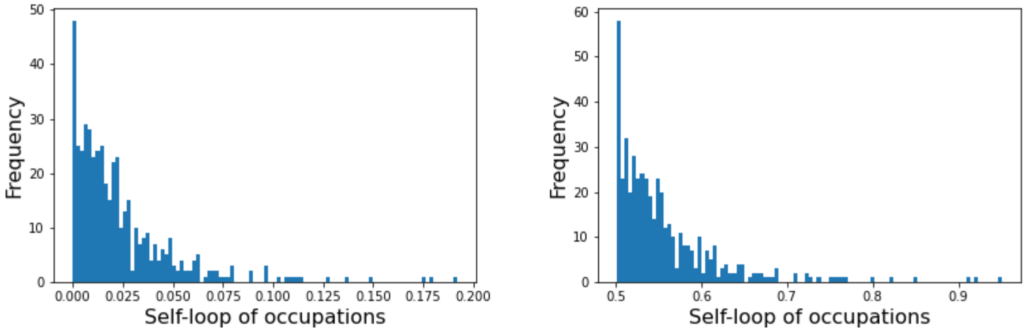

Figure S10: **Occupation's self-loop distribution** *Left* Self-loops of occupations before setting minimum value and linear re-scaling. *Right* Self-loop of occupations after setting minimum value and re-scaling.

S4.2 Parameter calibration robustness tests and validation discussion

In our calibration exercise of the Beveridge curve, we have four free parameters (δ_u, δ_v, τ, and a) and relatively few empirical observations (unemployment and vacancy rate), raising concerns of overfitting. However, our primary goal is not to show that we have a good fit for the Beveridge curve but to study the effects of limited occupational mobility on the automation-unemployment relationship. To show that the results on automation shocks are relatively immune to this issue of over-fitting, here we propose an alternative way to fit parameters δ_u and δ_v, one that does not depend on the Beveridge curve. We show that our main results hold for these new parameters.

Alternative calibration As shown in Fig. S8, the parameters δ_u and δ_v have a large effect on the shape and area enclose by the Beveridge. To address concerns that we are over-fitting the Beveridge curve, we propose a more simple calibration of parameters δ_u and δ_v that does not depend on the Beveridge curve. Instead of using the time series of vacancy and unemployment rate, we calibrate the model so that when we run it using a complete network, the unemployment and vacancy rate is 4%.

When we run the model using a complete network with parameters $\delta_u = \delta_v$, we can analytically derive the unemployment rate (see section S2.3.3). It follows from Eq. (S69) that the

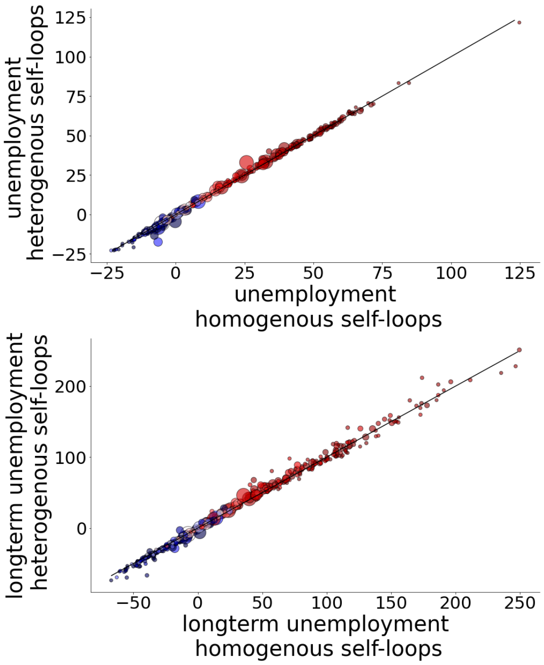

Figure S11: **Heterogenous self-loops effect** We compare the model results between using homogenous and heterogenous self-loops, for both percentage change in unemployment and long-term unemployment change

unemployment and vacancy rate equal 4% when $\delta_u = \delta_v = 0.026$. We then run the model as before but using parameter values $\delta_u = \delta_v = 0.026$. These values are approximately twice the original size. However, as we show in Fig. S12 our results remain robust. On the left we see that the increase in unemployment due to automation is larger for the occupational mobility network. On the right we see that the impact of automation on occupations varies substantially due to the network structure.

Overall there is a strong correlation between the unemployment and long-term unemployment changes for both calibrations at the occupation level. In Fig. S13 we show this correlation, where we see that all points lie close to the identity line. We also highlight the result for particular occupations. For both the original and alternative calibration, Childcare workers experience an increase in long-term unemployment (of 6% and 10% respectively), and Statistical technicians experience a decrease in long-term unemployment (of -24% and -18% respectively). This result shows that even though our original calibration may over-fit the Beveridge curve, the estimates of the impact of automation on employment are robust. That is, both calibration methods suggest that Statistical technicians are less likely to be long-term unemployed due to an automation shock than Childcare workers.

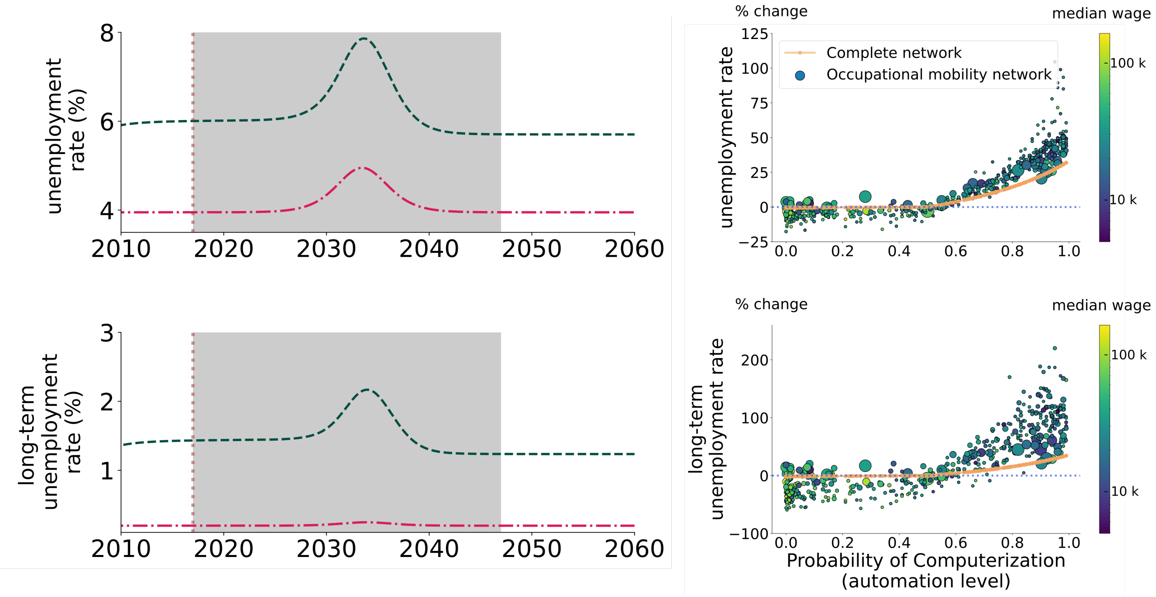

Figure S12: **Alternative calibration results** *Left* Unemployment and long-term unemployment rate using the alternative parameter values for δ_u and δ_v. *Right* The percentage change in unemployment and long-term unemployment for different occupations.

Results for different values of γ When target demand and realized demand differ, the difference between the two is reduced by a fraction γ of the difference, either by opening vacancies or separating workers, depending on the difference's sign. We expect that $\gamma \geq \delta_u$ and $\gamma \geq \delta_v$, so that market adjustment dynamics dominates random events. In the main text, we use $\gamma = 10\delta_u$ as a reference point. In this section, we explore how the results change for different values. In particular we test for $\gamma = 5\delta_u$ and $\gamma = 20\delta_u$. We chose these ranges since there is little change in the results for larger values of γ, and for lower values, we obtain unreasonably high values of the unemployment rate at the aggregate level (more than 15%).

In Fig. S21 we plot the percentage change in the unemployment rate using $\gamma = 10\delta_u$ (our benchmark) vs the percentage change in unemployment rate when $\gamma = 5\delta_u$ and $\gamma = 20\delta_u$ respectively. Our results show that the changes are very similar, although as γ increases, so does the increase in unemployment and long-term unemployment for occupations that are likely to be automated. These results are not surprising since the larger γ is, the faster the target demand's adjustment and thus sharper the shock.

Occupation-specific calibration and validation methods For simplicity, we used only the aggregate unemployment and vacancy rate to calibrate the model. However, the Great Recession likely had effects of different magnitude in each occupation. One could improve the calibration

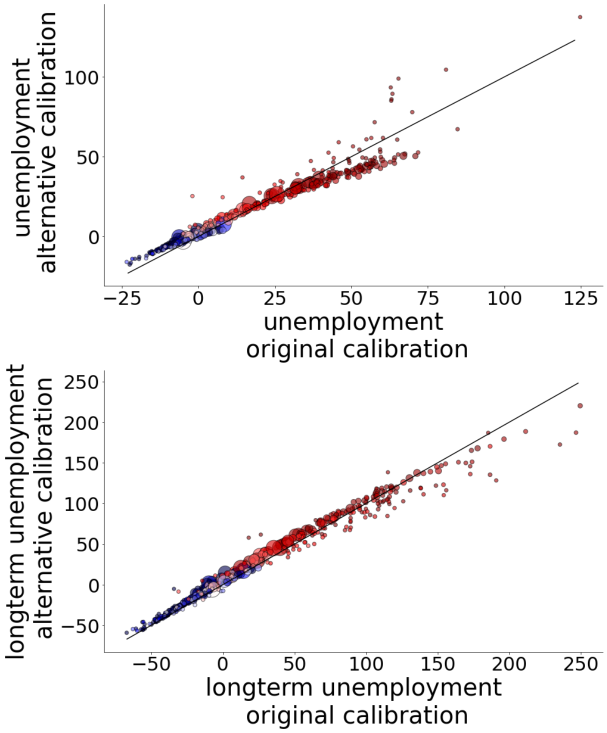

Figure S13: **Original and alternative calibration results comparison**. We plot the percentage change in unemployment (top) and long-term unemployment (bottom). The horizontal axis corresponds to the original calibration, the vertical axis to the alternative calibration.

method by using occupation-specific employment and vacancy levels. Although there is no publicly available vacancy data directly available at the occupation level, under some assumptions and using a couple of crosswalks, one could map industry vacancy data from the U.S. Bureau of Labor Statistics [6] into the 464 occupations used in this work. With this occupation-level data, one could match the target demand used for calibration to match the empirical demand distribution at the occupation level.

One could also use the occupation-level data to validate model predictions. Although this model's current aim is to show that the network structure plays an important role in understanding the impact of automation on occupational employment, we believe that (with substantial further work) one could develop a data-drive network model to forecast occupation-specific employment levels. To do this, one would need shock predictions that could be introduced into the model to calculate second-order effects. After that, one could use the occupation-specific data to validate such a model.

S4.3 Brynjolfsson et al. shock

Brynjolfsson et al. took a different approach than Frey and Osborne to assess the automatability of occupations. Taking advantage of the 8-digit level O*NET classification of occupations based on work activities [1], which has 974 occupations, they asked workers from a crowdsourcing platform to rate what they called the *suitability for machine learning* of each work activity. They then used the breakdown of work activities for each occupation to estimate the suitability for machine learning for each occupation. The Suitability for Machine Learning score is based on a five-point scale [2]. We normalize this measure by dividing it by 5 so that it is in a range from zero to one. Most occupations have at least some tasks that are suitable for machine learning, but few, if any, have all tasks suitable for machine learning. This suggests that many jobs will be re-designed rather than destroyed.

The Brynjolfsson et al. study yielded substantially different results than the Frey and Osborn study. First, these studies differ in their correlation to wages. The Frey and Osborne estimates have a strong negative correlation with wages, whereas the Brynjolfsson et al. estimates have a low correlation with wages. Second, as we see in Fig.1A, the distribution of the Frey and Osborne estimates is wide, whereas the Brynjolfsson et al. distribution has a narrow peak (see Fig.S14A). Since the Frey and Osborne estimates vary substantially between occupations and the Brynjolfsson et al. estimates, do not, the corresponding changes in the target labor demand are large for the Frey and Osborne shock but small for the Brynjolfsson et al. shock. (See Fig.4B and S15B for examples of how the target labor demand changes for different occupations under the two shocks).

[6] https://www.bls.gov/news.release/jolts.t01.htm

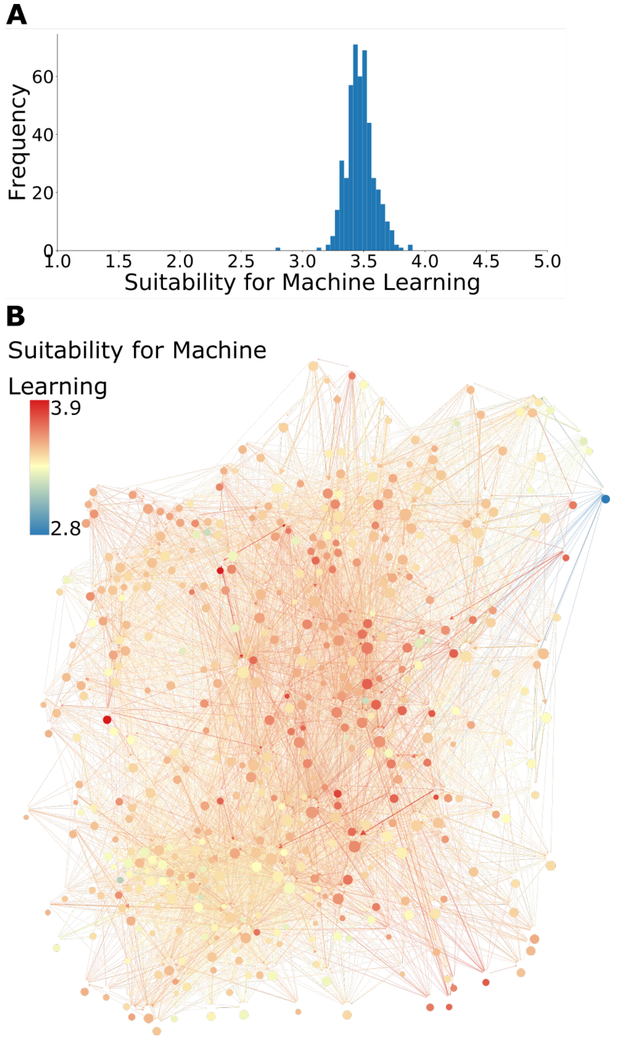

Figure S14: **Estimates of automatability in the occupational mobility network.** Panel (**A**) is a histogram of the suitability for machine learning as estimated by Brynjolfsson et al. [2]. Unlike the Frey and Osborne distributions, the suitability for machine learning distribution is unimodal. Panel (**B**) shows the occupational mobility network, where nodes represent occupations and links represent possible worker transitions between occupations. The color of the nodes indicates the suitability for machine learning. Red nodes have higher suitability for machine learning and blue nodes have a low one. The size of the nodes indicates the logarithm of the number of employees in each occupation.

The differences between the Frey and Osborne and the Brynjolfsson et al. shock imply that these shocks have a different effect on employment. The Brynjolfsson et al. shock causes no noticeable change in the aggregate unemployment or long-term unemployment rate (see Fig. S15B and C). This is because the Brynjolfsson et al. shock implies small changes in the target demand of occupations (for example, see Fig. S15A).

Although there is no noticeable change in the aggregate unemployment rates, the Brynjolfsson et al. shock still affects occupations disproportionately. This effect depends not only on the suitability for machine learning but also on each occupation's network position. As we observe in Fig. S15, the change in the long-term unemployment and unemployment varies substantially for occupations with similar suitability for machine learning. For example, both machinists and avionic technicians have a high 0.70 suitability for machine learning score. Still, long-term unemployment for machinists slightly increases, while the long-term unemployment for avionic technicians *decreases* by more than 20%. In other words, our results suggest that retraining efforts would be better spent on machinists than on avionic technicians.

S4.4 Automation time and adoption rate

This section discusses how our results change when we assume a different duration of the automation shock. We assume that automation happens within 20 or 40 years, instead of 30. We measure the change in unemployment during the whole automation period and during the *steep* transition period. We define the steep transition period as the middle part of the automation period when the sigmoid is steepest. In Fig. S17 we highlight the whole automation period with a grey area coloring and the steep automation period by a coral shadowing.

As expected, the shorter the automation period is, the larger the increase in the aggregate unemployment and long-term unemployment rates (see top panels of Fig. S17). On the bottom panels of Fig. S17 we plot the percentage change in the unemployment and long-term unemployment rates of each occupation during the whole automation period vs. the percentage change of the unemployment rates of each occupation during the steep automation period. There is a strong correlation between unemployment change during the whole transition period and the steep transition period. However, during the steep automation period, the unemployment rate's percentage change is more extreme than during the entire automation period. Namely, occupations with high automation levels have a higher percentage change in the unemployment and long-term unemployment rate during the steep automation period than during the whole automation period. Likewise, occupations with low automation levels tend to decrease their unemployment and long-term unemployment rate more during the steep automation period than over the entire automation period.

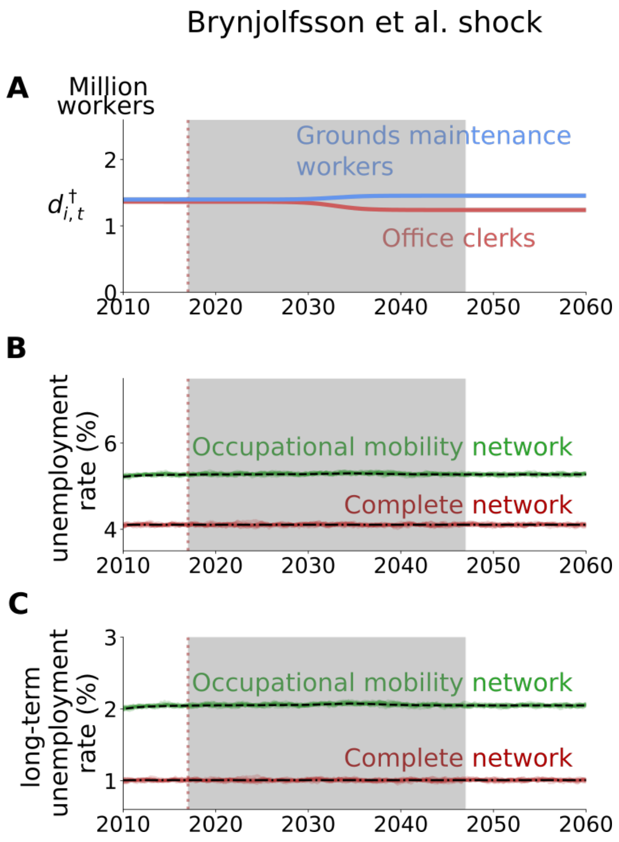

Figure S15: **Aggregate labor market outcomes under the Brynjolfsson et al. shock.** The grey area denotes the 30 years during which the automation shock takes place. Panel (**A**) shows the evolution of the target labor demand for two example occupations. The occupation colored in blue has a low suitability for machine learning and the occupation colored in red has a high one. Because the distribution of the suitability for machine learning is more evenly distributed across occupations that the probability of computerization, the Brynjolfsson et al. shock implies a small change in the target labor demand of most occupations. Panel (**B**) shows the unemployment rate as a function of time. Dashed lines are our approximations of the expected value (solved numerically) and the solid lines are 10 simulations with 1.5 M agents. Panel (**C**) shows the long-term unemployment rate as a function of time. As before, dashed lines correspond to the deterministic approximation of Eqs. (12 - 14) and solid lines to the full stochastic model simulation of Eqs. (2 - 4).

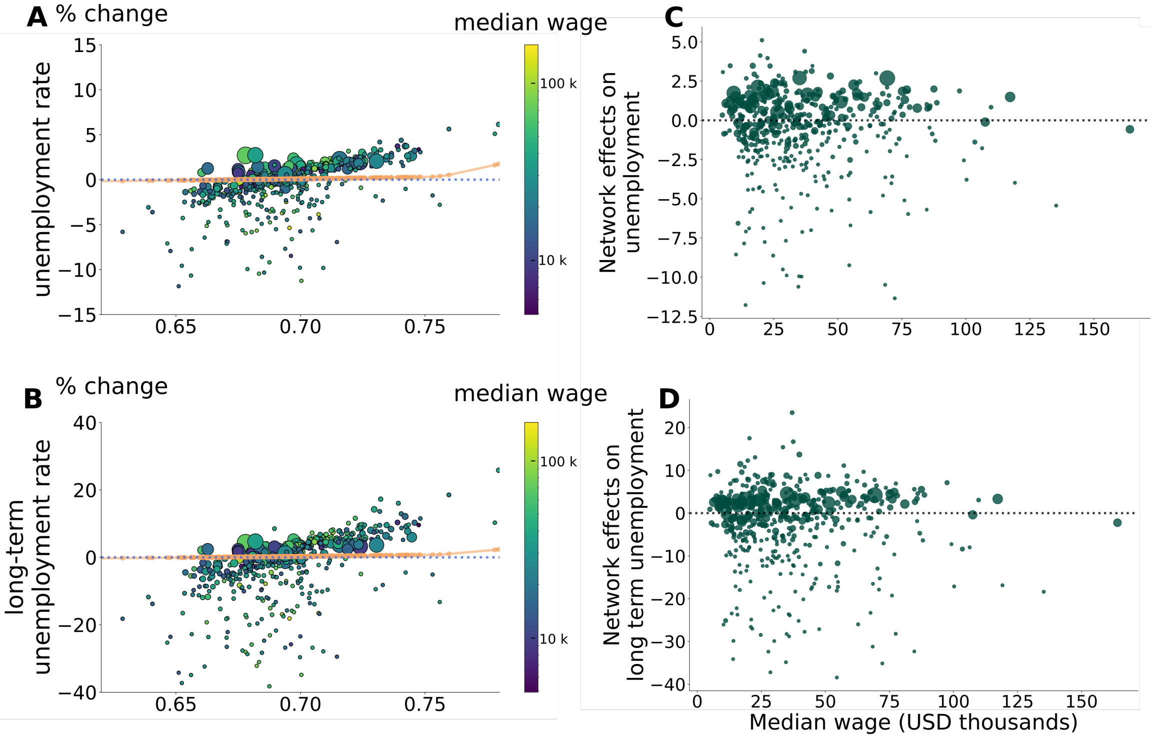

Figure S16: **Impact of the Brynjolfsson et al. shock on unemployment and long-term unemployment at the occupation level.** The green dots are for the occupational mobility network and the red dots are for the complete network. The size of the green dots is proportional to the employment of the occupation they represent. Panel **(A)** shows the percentage change in the unemployment rate vs the automation level for each occupation, while panel **(B)** shows the same thing for the long-term unemployment rate. The scatter in the results demonstrates that, due to network effects, the automation level only partially explains occupational unemployment. The right panels show the network effects i.e. the difference between the green and the black dots in the left panels. If this difference is positive, network effects are detrimental for the occupation. Panel **(C)** Network effects on percentage change in unemployment rate vs. median wage. Panel **(D)** Network effects on percentage change in long-term unemployment rate vs. median wage.

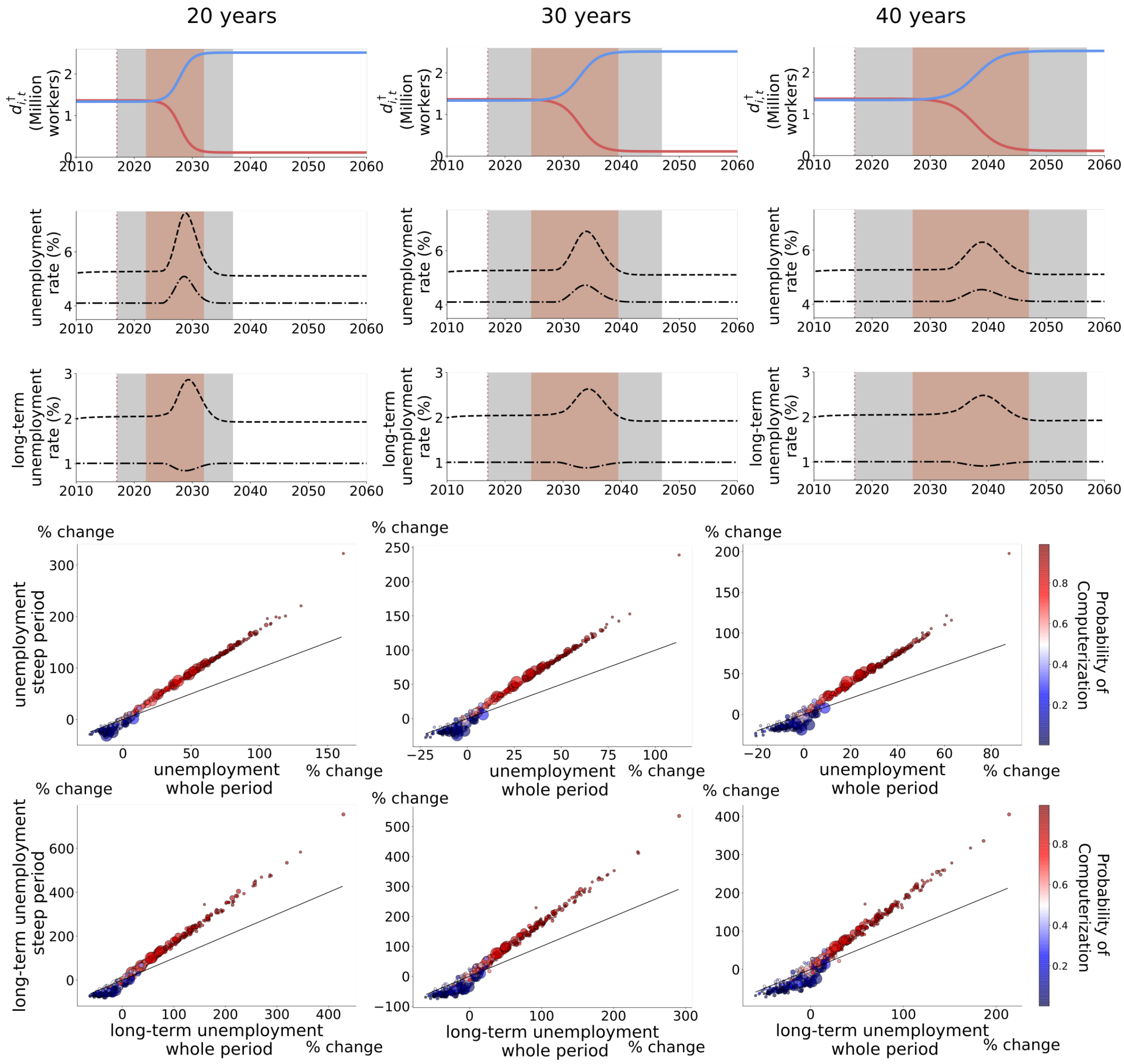

Figure S17: **Effect of shock duration and length of the measuring window on the measurements of unemployment rates Top.** For different duration of the automation shock (20, 30 and 40 years) we show the target demand of two occupations with similar demand level (Childcare workers and officer clerks), the unemployment, and long-term unemployment rates. The grey area denotes the whole period of automation, meaning that the target demand has reached the automation level within a 1×10^{-4} tolerance. The coral area denotes the sharp transition period which is middle steepest part of the sigmoid shock. **Bottom.** For each occupation we plot the percentage change during the whole transition period vs the percentage change during the sharp transition period. Occupations are colored by their automation probability.

S4.5 Automation shocks that change aggregate labor demand

We assumed that the aggregate demand remains constant after the shock. In this section, we relax this assumption, and we run the model for an aggregate demand increasing or decreasing by 5%. We use the Frey and Osborne automation shock.

Fig. S18 shows scatter plots of the change in (automation period) unemployment rates when aggregate labor demand is constant vs. when it changes. As expected, when the aggregate demand increases, the percentage change in unemployment and long-term unemployment is lower, and the points lie below the identity line. When the aggregate demand decreases, the percentage change in unemployment and long-term unemployment is larger, and the points lie above the identity line. In contrast, there is a strong correlation between the changes in the unemployment rates when the demand changes. When the demand remains constant, occupations with low automation probabilities (blue dots) lie further away from the identity line. This result means that the structural part of the automation shock mostly affects the occupations with high automation estimates. When we include a change in the aggregate labor demand, then occupations with low automation estimates are also affected considerably.

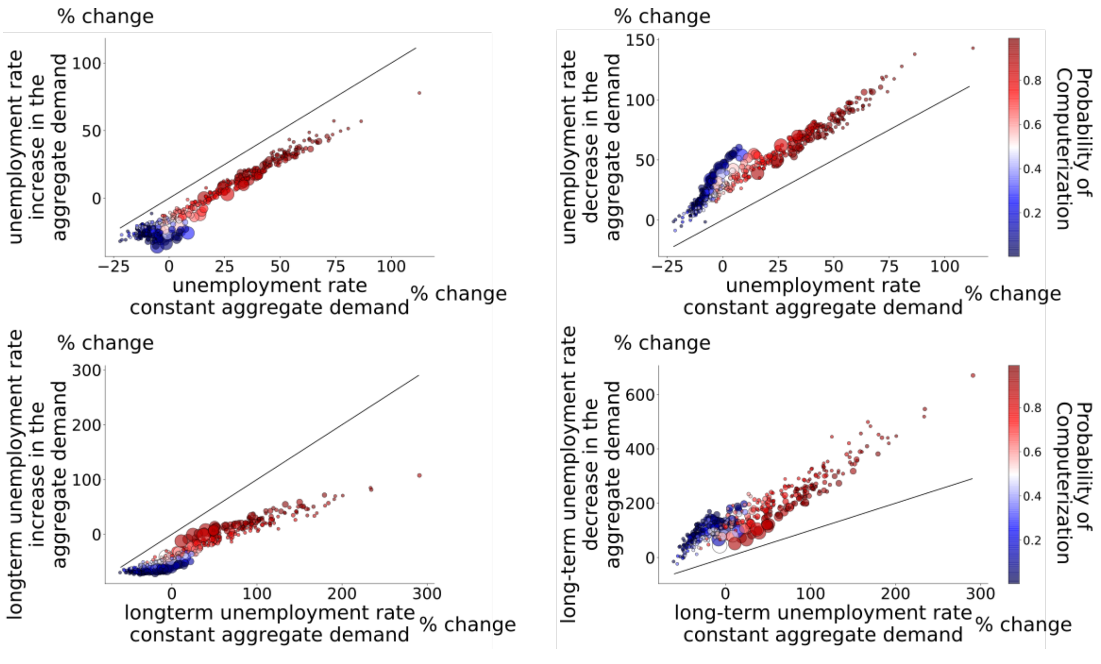

Figure S18: **Frey and Osborne shock with different post-automation target demand scenarios**. In each panel we plot on the x-axis the percentage change in the period unemployment rates when the aggregate demand does not change and on the y-axis the percentage change in the period unemployment rate when the aggregate demand does change. On the left panels we assume the aggregate demand increases by 5% and on the right we assume it decreases by 5%.

S4.6 A different measure of unemployment and long-term unemployment during automation

In the main text, we define the occupation-specific average unemployment and average long-term unemployment as

$$u_{i,\text{average}}(T) = \frac{100}{T} \frac{\sum_{t \in T} u_{i,t}}{\sum_{t \in T}(u_{i,t} + e_{i,t})}$$

and

$$u^{(\geq \tau)}_{i,\text{average}}(T) = \frac{100}{T} \frac{\sum_{t \in T} u^{(\geq \tau)}_{i,t}}{\sum_{t \in T}(u_{i,t} + e_{i,t})}.$$

However, we could have chosen to compute the unemployment rate at each time step of the automation period and then take the average, that is

$$u_{i,\text{alternative}}(T) = \sum_{t \in T} \frac{u_{i,t}}{(u_{i,t} + e_{i,t})}$$

and

$$u^{(\geq \tau)}_{i,\text{alternative}}(T) = \sum_{t \in T} \frac{u^{(\geq \tau)}_{i,t}}{(u_{i,t} + e_{i,t})}.$$

In Fig. S21 we compare the change in the average unemployment and long-term unemployment rates with the change in the alternative unemployment and long-term unemployment rates. On the top-right, we show the change in the average unemployment rate in green and the change in the alternative unemployment rate in cyan. On the bottom-right, we do the same for the long-term unemployment rate. Both these plots show that there is a substantial overlap between the average change and the alternative change.

We plot the change in the average unemployment rate vs. the alternative unemployment rate on the top-left panel for better visualization. We plot the change in the average long-term unemployment rate vs. the alternative long-term unemployment rate on the bottom-left. We observe that almost all occupations lie close to the identity line, except occupations with low employment (small circles) and are highly likely to be automated (red color). These occupations, which are highly automatable and have low employment, substantially increase their unemployment and decrease their employment share (due to the structural change). Thus, the ratio between the two, which is considered by the alternative unemployment rates, increases considerably. In contrast, when we measure the average unemployment rate, the initial employment share prevents a sharp increase. However, both measurements exhibit the network effects – occupations with similar automation probabilities have different percentage changes in their unemployment and long-term unemployment rates.

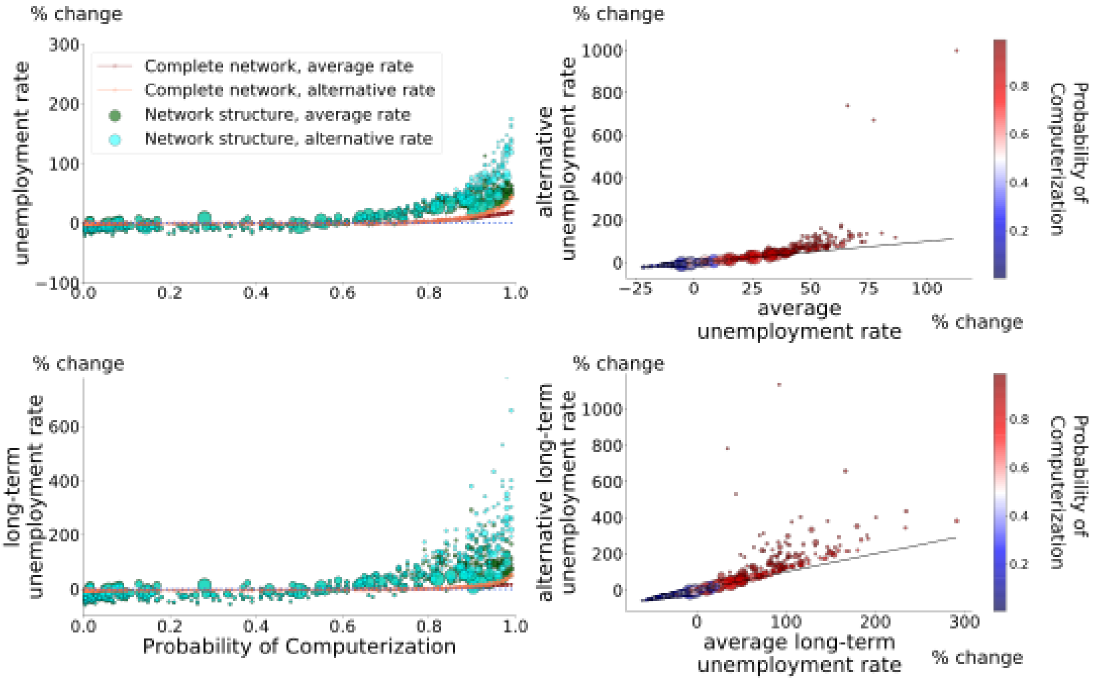

Figure S19: **Average and alternative unemployment and long-term unemployment rates Left.** Percentage change in the average unemployment and long-term unemployment rates in green and the percentage change in the alternative unemployment and long-term unemployment rates in cyan. **Right.** Percentage change of the average unemployment and long-term unemployment rate vs the percentage change of the alternative unemployment and long-term unemployment rate

Figure S20: Expected change in unemployment and long-term unemployment with different γ parameters.

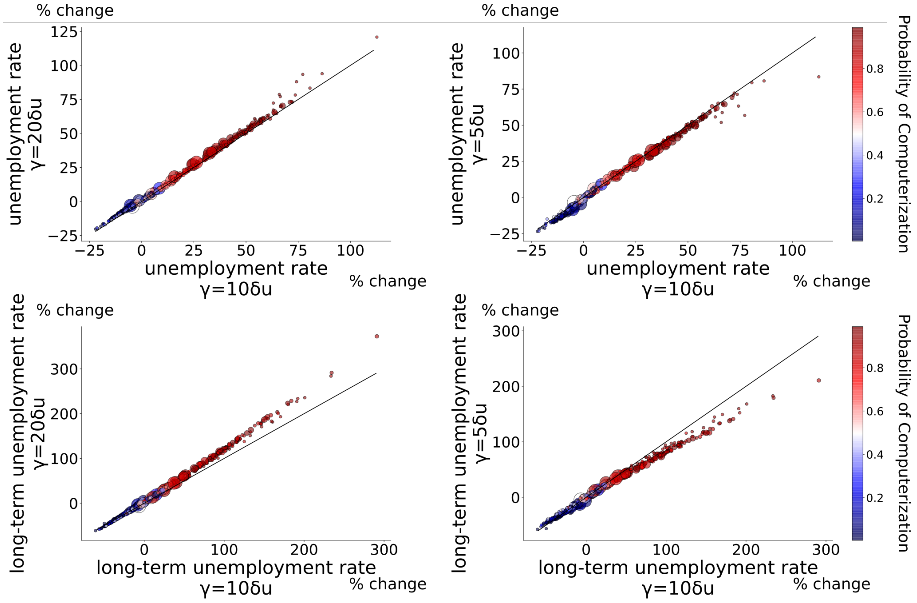

Figure S21: **Change in unemployment and long-term unemployment with different values of gamma** Top panels show the change in unemployment rates vs the automation probability. The bottom panels show the change when $\gamma = 5\delta_u$ and $\gamma = 20\delta_u$ on the y axis and on the x-axis when $\gamma = 10\delta_u$.

S5 The network structure, retraining effects, and steady-state shifts

This section further explores how the network structure affects the impact of automation on employment with three exercises. In the first exercise, we test our conjecture that the steady-state decrease in unemployment after automation happens because the automation shock distribution across the network structure is assortative. In the second exercise, we study random network structures that preserve some of the occupational mobility network's statistical features. Finally, we explore how a retraining policy could affect the network structure and mitigate the impact of automation on employment.

S5.1 Shift in the steady-state unemployment post-automation

In this section, we give arguments that support our conjecture that the Frey and Osborne shock causes such persistent effects since automation levels of neighboring occupations tend to be similar. In other words, the distribution of the automation level across occupations is assortative in the occupational mobility network.

To test our conjecture, we create a surrogate Frey and Osborne shock by randomizing occupations' automation levels. We do this by randomly shuffling the automation level of each of the 464 occupations, i.e., randomly reassigning each automation level to a new occupation (without replacement). This preserves the distribution of automation levels but removes any correlation between neighboring occupations, i.e., this breaks the assortative distribution of automation levels. When we do this, the aggregate unemployment rate does not decrease, while the long-term unemployment tends to increase slightly (see Fig.S22). Thus the most persistent effects disappear when the correlation inherent in the network structure is removed.

To show that the assortative distribution of automation levels in the occupational mobility network can cause the steady-state unemployment to decrease, we create another surrogate shock where we randomize relative to the Frey and Osborne shock while intentionally creating a correlation between neighbors. Since occupations of the same classification typically have high connectivity, we redistribute the probabilities of computerization so that occupations with similar classifications have a similar automation level. We do this by ordering the probabilities of computerization in ascending order and ordering the occupations in ascending order with respect to their occupation code. We then match these to create a surrogate shock with the desired property. When we impose this shock, the post-automation aggregate unemployment, and long-term unemployment rate decrease, supporting our conjecture (see Fig.S22).

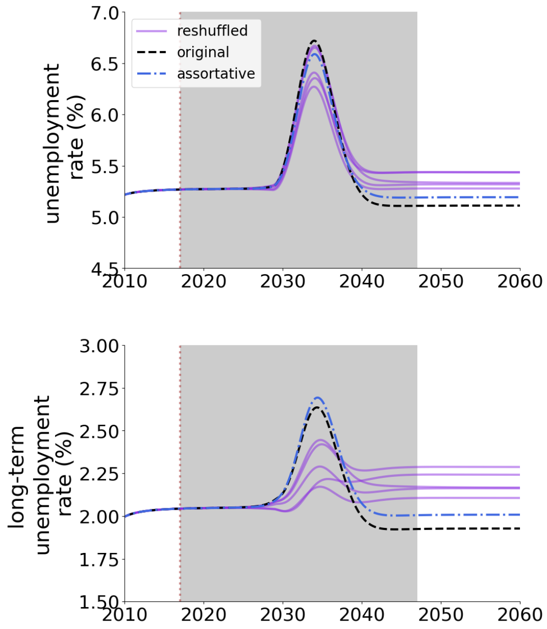

Figure S22: **Randomized and assortative versions of the Probability of Computerization shock.** On the top the unemployment rate. On the bottom the long-term unemployment rate.

S5.2 Randomizing the network structure

We randomize the network structure of the occupational mobility network in two ways: the *weight reshuffling* and the *edge rewiring*. We keep the edges fixed in the weight reshuffling but randomize the number of transitions between occupations and then renormalize to have a column-stochastic adjacency matrix. In the edge rewiring, we randomize the ending point of an edge and preserve the edge's weight and starting point. We do not need to renormalize the adjacency matrix since by keeping the starting point of the edge and its weight fixed, the column-sum of the adjacency matrix remains constant. These randomizations preserve different information about the network structure. The weight reshuffling preserves the topology, while the edge rewiring preserves the out-strength (i.e., weighted out-degree) but changes the topology. We run each randomization ten times and analyze how our results change.

At the aggregate level, both randomizations have a lower spike in unemployment and long-term unemployment during the automation shock (see Fig. S23). This is expected given that, in the occupational mobility network, occupations with high automation probability tend to be clustered together. Randomizing the structure destroys clustering and allows an easier transition into the new steady state.

As shown in Fig. S23 the reshuffling of weights (blue lines) shows a much larger decrease

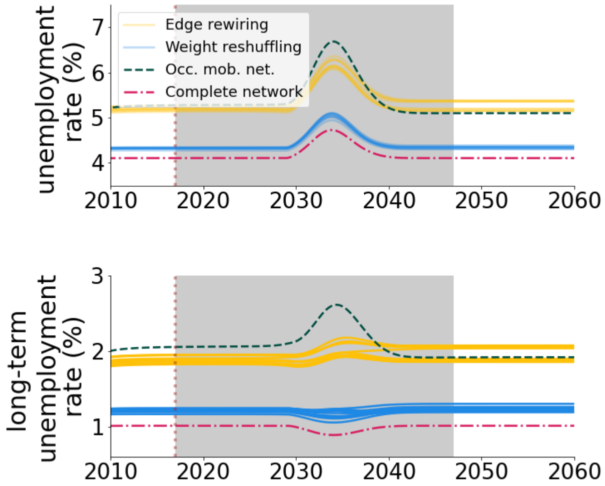

Figure S23: **Network structure effect at the aggregate level** We show the change in unemployment and long-term unemployment due to the automation shock for different networks. The increase in unemployment rates is larger for the occupational mobility network. Both randomizations decrease the automation spike in unemployment rates, the weight reshuffling substatially more.

in unemployment and long-term unemployment than the rewiring of edges (yellow lines). This happens because most of the information on the occupational mobility network is in the weights. The occupational mobility network is dense; the mean degree of a node is 155. In other words, on average, there has been at least one worker of each occupation transitioning into 155 out of 464 other occupations. The median degree of a node is 134, which is close to the mean. However, the number of transitions encoded in the weights vary substantially. When we randomize the weights, we lose the most significant part of the structure, while rewiring edges does not destroy too much information.

At the disaggregated level, occupations experience a considerably lower increase in unemployment and long-term unemployment percentage change in the randomized networks than in the occupational mobility network. In Fig. S24, we compare the percentage changes in the unemployment rates when we run the model using the occupational mobility network and one realization of the reshuffled version. The black line is the identity, and the green line the linear fit. In all panels, the slope of the green line is noticeably lower than one. In other words, the change in unemployment rates for occupations is substantially less for the randomized versions of the occupational mobility network. The difference is more pronounced for long-term unemployment. This makes sense since the unemployment change is driven to a larger extent by the automation shock, while the network structure has a higher effect on long-term unemployment.

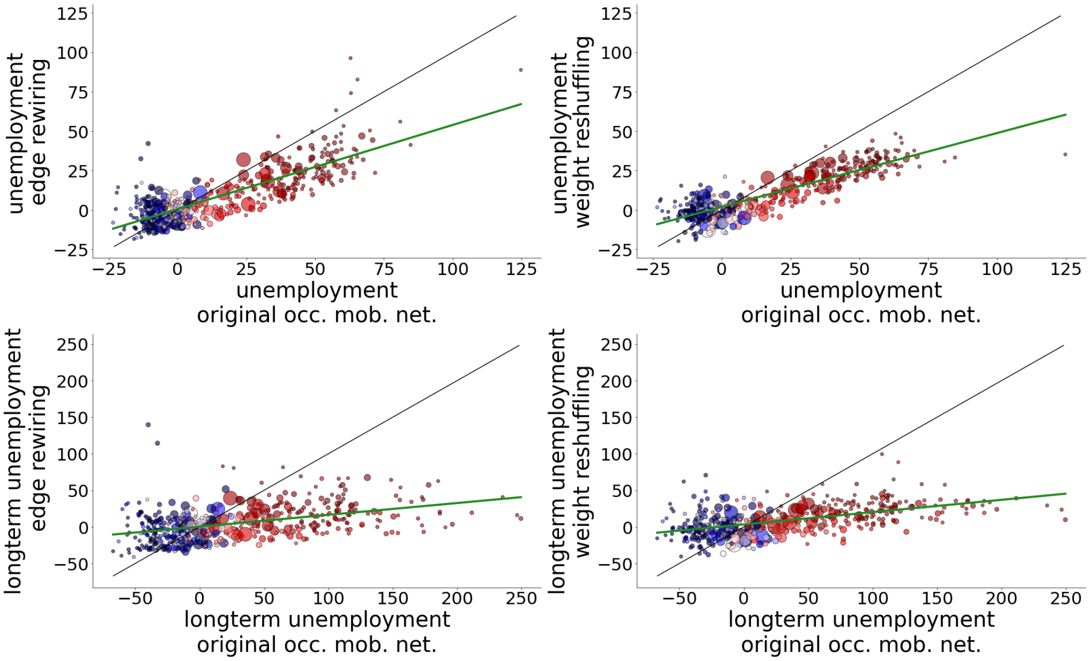

Figure S24: **Randomized network structure comparison with original network results**. On the top panels we plot the percentage change in unemployment when running the model using the OMN and the reshuffled version of the OMN. On the bottom panels we plot the percentage change in long-term unemployment comparison. On the left we show the egde reshuffling and on the right the weight reshuffling. In both scenarios the changes in the unemployment rate are considerably smaller for the reshuffled versions of the OMN.

S5.3 Retraining policies

We model retraining schemes by adding links to the occupational mobility network. Acknowledging that retraining is most plausible between somewhat similar occupations, we restrict the possible edges that we can add as follows. Previous research [8] has built the Job Space network, where occupations are linked according to the number of work activities they share (weighted by a scarcity factor). This research shows that sharing work activities is the best predictor of empirical occupation transitions. We consider a retraining scheme between occupations to be viable only if there is an edge between the two occupations in the Job Space with a weight above $\iota_{js} = 0.0013$, which is roughly half the mean weight of the Job Space network.

The first retraining scheme we consider focuses on the subset of occupations that experience a high increase in long-term unemployment (above 50%). We link these occupations to ten occupations that experience lower long-term unemployment. We choose these ten occupations according to the weight of the edge connecting them in the Job Space. For all the subset of high unemployment occupations, we are able to find ten low unemployment occupations that have a link with the high unemployment occupation with weight above ι_{js}. Once we selected the ten low unemployment occupations for each of the high unemployment occupations, we add edges with a weight equal to $\iota_{omn} = 0.006$, which is the mean weight of the edges in the occupational mobility network. Finally, we renormalize the occupational mobility network with the added retraining links so that it is column-stochastic since we use these values as transition probabilities.

The second retraining scheme we consider is random. We add the same number of links as in the previous exercise, but we select occupation pairs randomly. We keep the restriction of only adding edges with a weight larget than ι_{js} in the Job Space and also add them with a weight of ι_{omn} in the occupational mobility network. As before, we also renormalize so that the matrix is column-stochastic.

Our results show that both the targeted retraining random retraining decrease the spike in unemployment and long-term unemployment caused by automation at the aggregate and occupational level (see Figs. S25 and S26). Similar to what happened for the occupational mobility network, the post-automation steady-state unemployment rate is lower than the pre-automation unemployment rate for the networks with retraining (see Fig. S25). The random retraining decreases the aggregate unemployment rate slightly more than the targeted retraining policy. This is likely because the occupations whose long-term unemployment increased the most tend to have low employment. Therefore targeted retraining may be targeted to occupations with few workers and thus involve fewer workers than the random retraining overall, which is shown in the aggregate level post-automation steady-state.

It might still be surprising that the random retraining works so well at the disaggregated level. This is another result that highlights the importance of the network structure. Even if

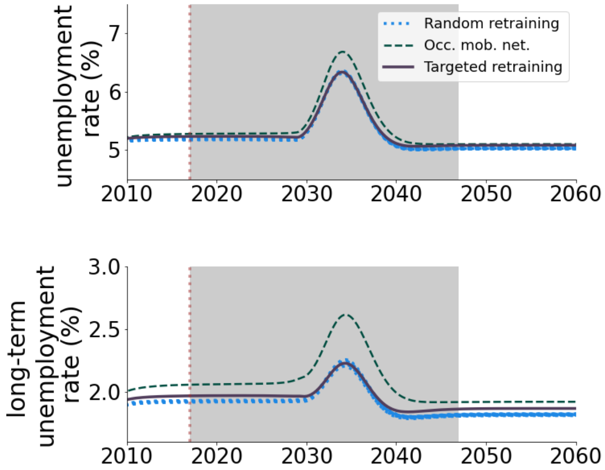

Figure S25: **Retraining policies** *Left* The aggregate outcome of retraining policies, for either random retraining or targeted retraining. In blue, we plot 10 realizations of the random retraining strategy, all show very similar outcomes.

retraining is not directly targeted towards the most affected occupations, increasing mobility helps the affected occupations through second-order effects by freeing vacancies in adjacent occupations. It is important to bear in mind that we do not consider wage dynamics, which would play an important role for workers being harshly affected.

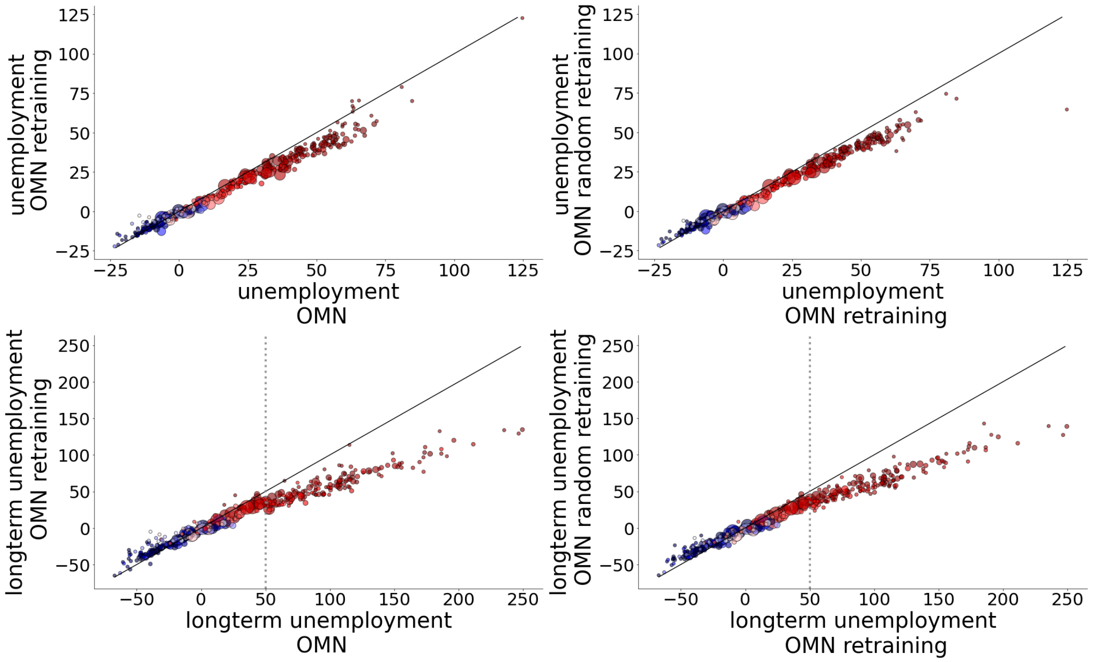

Figure S26: **Retraining policies at the occupation level** *Left* Targeted retraining towards occupation with long-term unemployment percentage change above 50 *Right* Random retraining. We pick one at random one realization of the random retraining to show in this plot.

References

[1] Erik Brynjolfsson and Tom Mitchell. What can machine learning do? workforce implications. *Science*, 358(6370):1530–1534, 2017.

[2] Erik Brynjolfsson, Tom Mitchell, and Daniel Rock. What can machines learn, and what does it mean for occupations and the economy? In *AEA Papers and Proceedings*, volume 108, pages 43–47, 2018.

[3] Sarah Flood, Miriam King, Renae Rodgers, Steven Ruggles, and J. Robert Warren. Integrated public use microdata series, current population: Version 8.0 [dataset], 2020. Minneapolis, MN: IPUMS, 2020., `https://doi.org/10.18128/D030.V8.0`.

[4] Morgan R Frank, David Autor, James E Bessen, Erik Brynjolfsson, Manuel Cebrian, David J Deming, Maryann Feldman, Matthew Groh, José Lobo, Esteban Moro, et al. Toward understanding the impact of artificial intelligence on labor. *Proceedings of the National Academy of Sciences*, page 201900949, 2019.

[5] Carl Benedikt Frey and Michael A Osborne. The future of employment: how susceptible are jobs to computerisation? *Technological Forecasting and Social Change*, 114:254–280, 2017.

[6] Fane Groes, Philipp Kircher, and Iourii Manovskii. The u-shapes of occupational mobility. *The Review of Economic Studies*, 82(2):659–692, 2014.

[7] Jeffrey Lin. Technological adaptation, cities, and new work. *Review of Economics and Statistics*, 93(2):554–574, 2011.

[8] Penny Mealy, R. Maria del Rio-Chanona, and J. Doyne Farmer. What you do at work matters: New lenses on labour. *SSRN 3143064*, 2018. Available at SSRN: https://ssrn.com/abstract=3143064 or http://dx.doi.org/10.2139/ssrn.314306.

[9] Valerie A Ramey and Neville Francis. A century of work and leisure. *American Economic Journal: Macroeconomics*, 1(2):189–224, 2009.

[10] Margaret Stevens. New microfoundations for the aggregate matching function. *International Economic Review*, 48(3):847–868, 2007.

[11] Paul Stoneman. *The economics of technological diffusion*. Wiley-Blackwell, 2001.
